# Supplementary material for: An experimental framework to assess biomolecular condensates in bacteria
Source: Nat Commun. 2024 Apr 15;15:3222. doi: 10.1038/s41467-024-47330-4 (PMC11018776; doi:10.1038/s41467-024-47330-4)
Supplement: Supplementary file 1 — Supplementary Information [file 41467_2024_47330_MOESM1_ESM.pdf]

An experimental framework to assess biomolecular condensates in bacteria

Authors

Y Hoang[1,4], Christopher A. Azaldegui[2,4], Rachel E. Dow[1], Maria Ghalmi[1], Julie S. Biteen[2,3*], and Anthony G. Vecchiarelli[1*]

Supplementary information

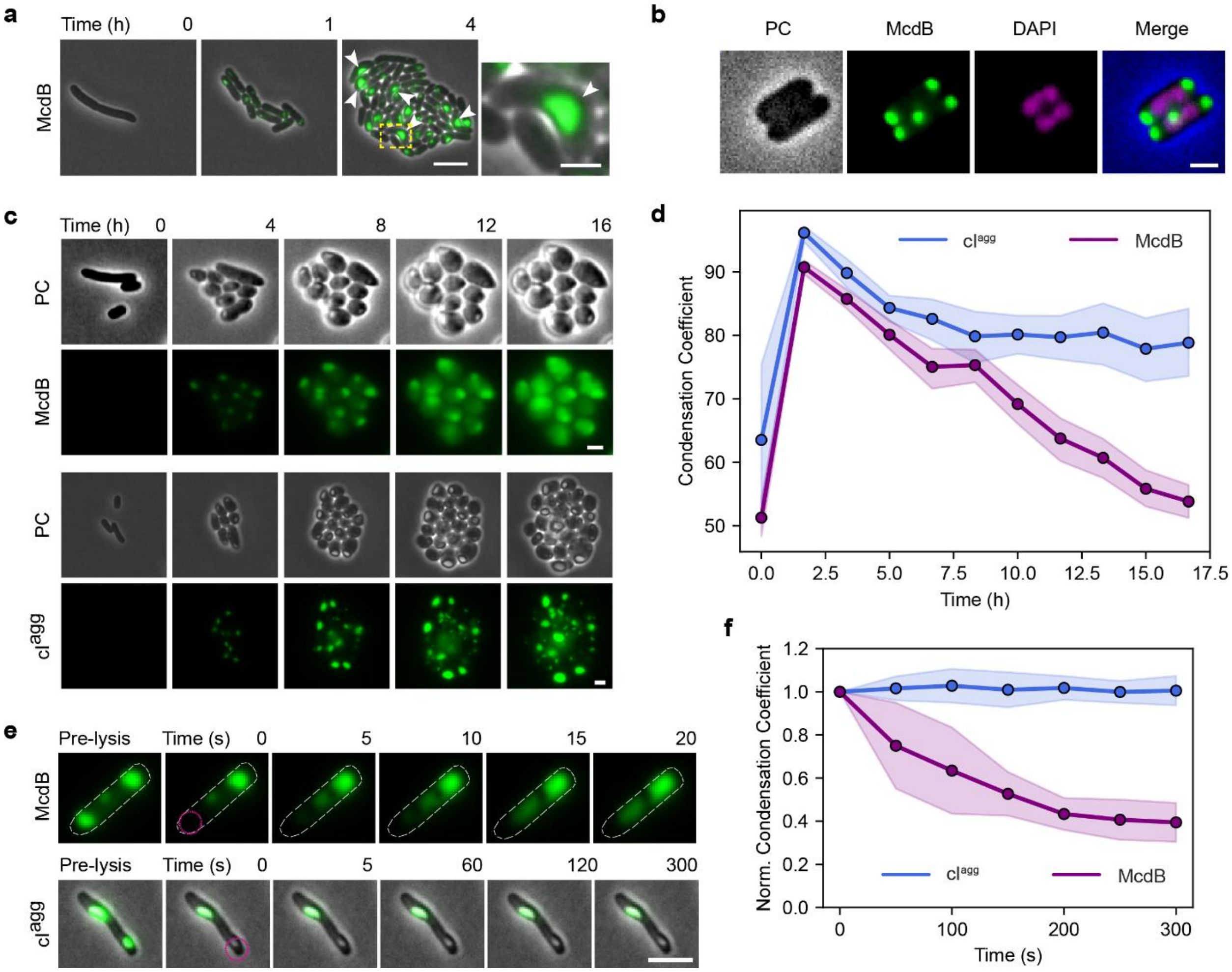

Figure S1. Overexpression of mNG-McdB in *E. coli* BL21. a. mNG-McdB forms foci. The phase contrast channel is shown in gray and the mNG channel is in green. Images are representative of three biological replicates. White arrows highlight cells where McdB foci wet to the inner membrane inducing cell curvature. Scale bars: 5 µm. **b**. mNG-McdB foci are nucleoid-excluded. DAPI stain is shown in magenta. Scale bar: 1 µm. **c.** Effects of changing cell volume on focus stability. *E. coli* cell volume was increased by treating cells with the MreB inhibitor, A22 (10 µg/mL). Images were taken at 6 h post-treatment, when rod-shaped cells transitioned their morphology into round cells. Scale bar: 5 µm. **d**. Quantification of cells shown in (c). Solid lines and shading represent the mean condensation coefficient of a representative cell population (n > 10 cells) and its 95% confidence interval. An intensity threshold of $I = 0.5$ was used; a condensation coefficient of 50 is equivalent to a homogenous pixel intensity distribution. **e**. Localized lysis of the cell. One cell pole was lysed using a high-intensity laser, focused within the indicated magenta circle. Representative frames show solubilization of the opposing focus and shift of fluorescent signal to the opposite end. Scale bar: 1 µm. **f.** Quantification of three representative cells (n = 3) for each protein shown in (e). Solid lines and shading represent the mean condensation of three representative cells and the standard deviation. An intensity threshold of $I = 0.1$ was used.

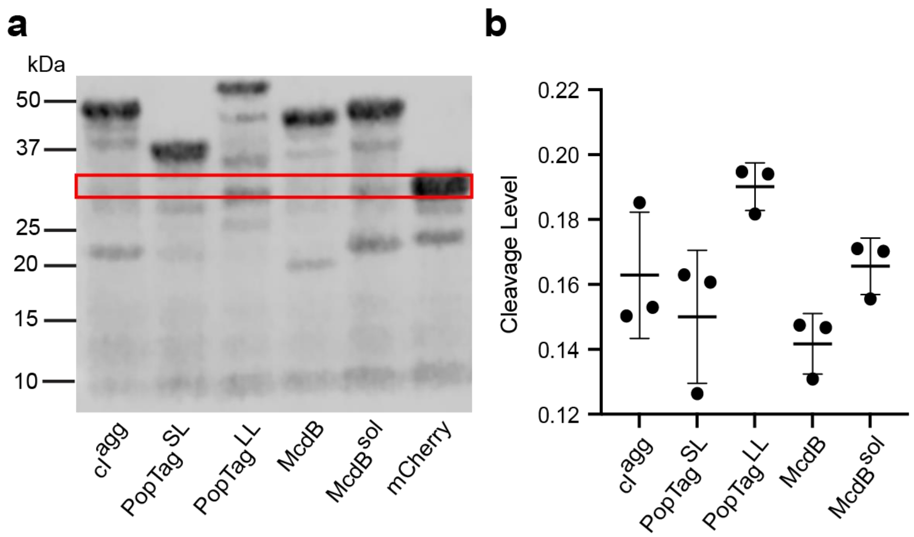

Figure S2. Expression of mCherry fusion proteins. *E. coli* MG1665 cells of indicated strains were induced with 1 mM IPTG for 2 h. **a**. Immunoblot analysis of mCherry fusion proteins. Representative immunoblots are shown. Red box shows the band at the mCherry size (28 kDa). Similar results were observed in three biological replicates. **b**. Cleavage level of mCherry fusion proteins. In each lane, the intensity of the band at the mCherry size was quantified and normalized to the total intensity of the respective lane. Error bars represent the standard deviation from the three biological replicates (n = 3).

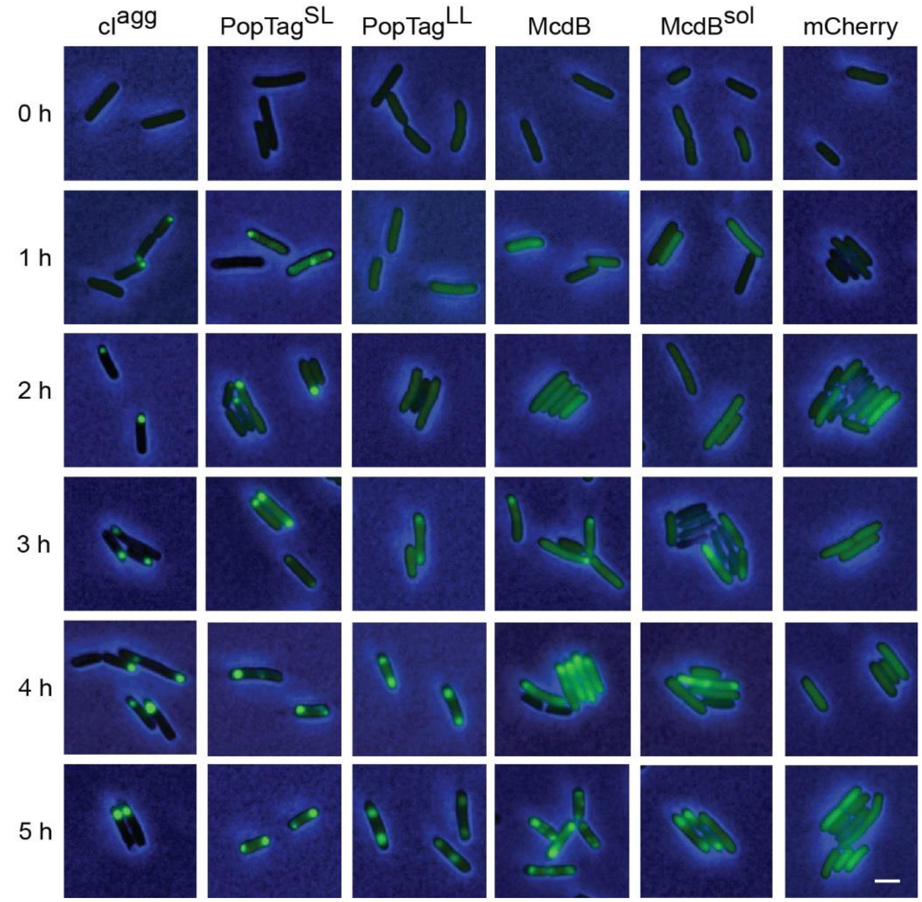

Figure S3. mCherry fusion proteins form cellular foci. Representative images of indicated mCherry fusion protein foci in *E. coli* from 0 to 5 h post-induction. Phase contrast (blue) and mCherry (green) channels were merged. Images are representative of three biological replicates (*n* = 3). Scale bar: 1 μm.

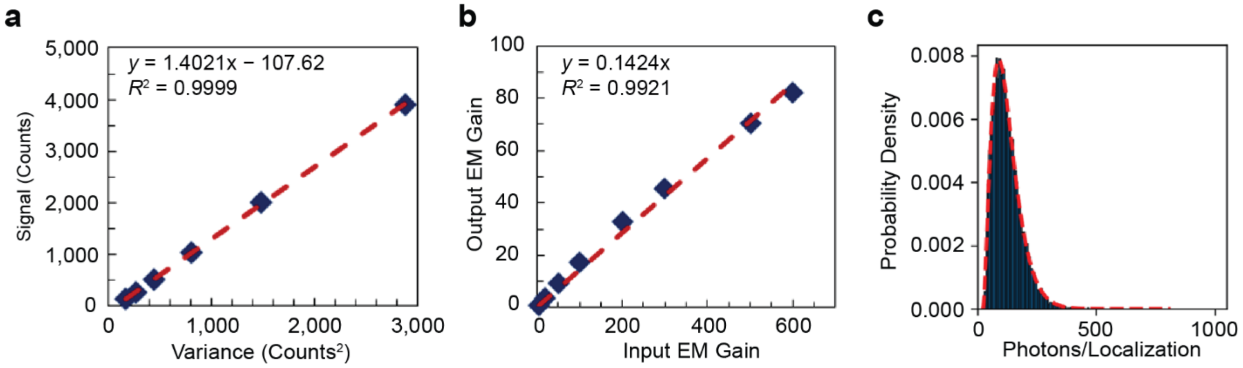

Figure S4. Electron-multiplying charge-coupled detector (EMCCD) calibration for photon counting. **a**. Signal variance versus signal average for different camera integration times. The data points correspond to the following camera integration times: 10, 20, 40, 80, 160, and 320 ms. The dashed red line is the linear regression. **b**. Electron multiplication conversion plot. The dashed red line is the linear regression constrained through the origin. **c**. Distribution of number of detected photons per localization per imaging frame for mCherry-McdB. Signal histogram was fit to a gamma distribution, determining a peak of 91.1 photons per localization at a 40-ms camera integration time. 35800 localizations were used to build the histogram.

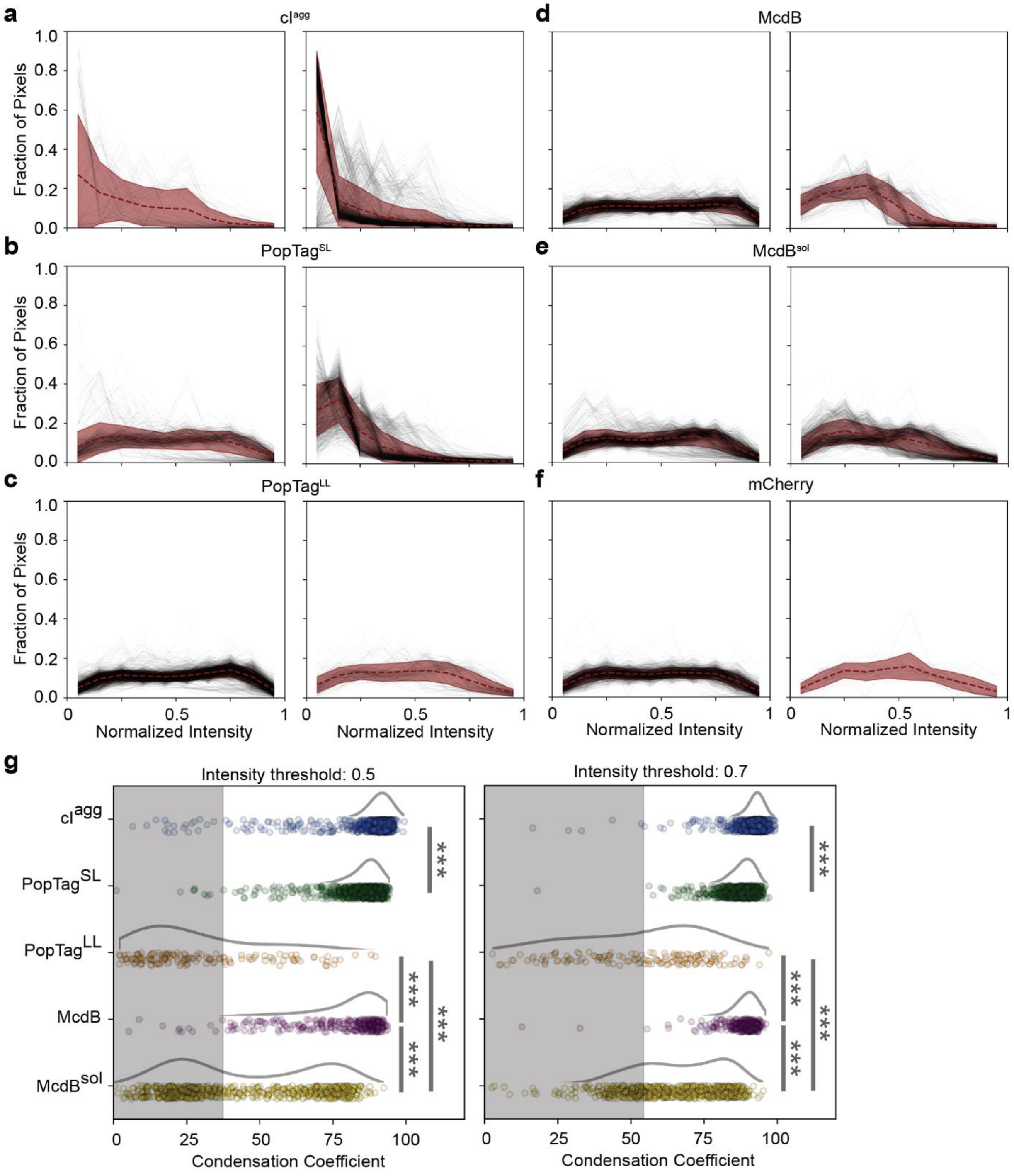

Figure S5. Normalized pixel intensity histograms and condensation coefficients of mCherry fusion proteins. a-f. Normalized pixel intensity histograms for cells with no detected focus (left) and a detected focus (right). Black lines represent individual cells. The red dashed line and shading are the average and standard deviation across cells for each plot. **g**. Quantification of condensation coefficients. Condensation coefficient plots for cells with a detected focus and threshold values of I = 0.5 and 0.7. Data points correspond to individual cells. The curves next to the scatter plots were obtained via kernel density estimation. The shaded region represents the measurement range for cells expressing a uniform mCherry signal. Analysis was done on N > 900 cells for each fusion over three biological replicates.

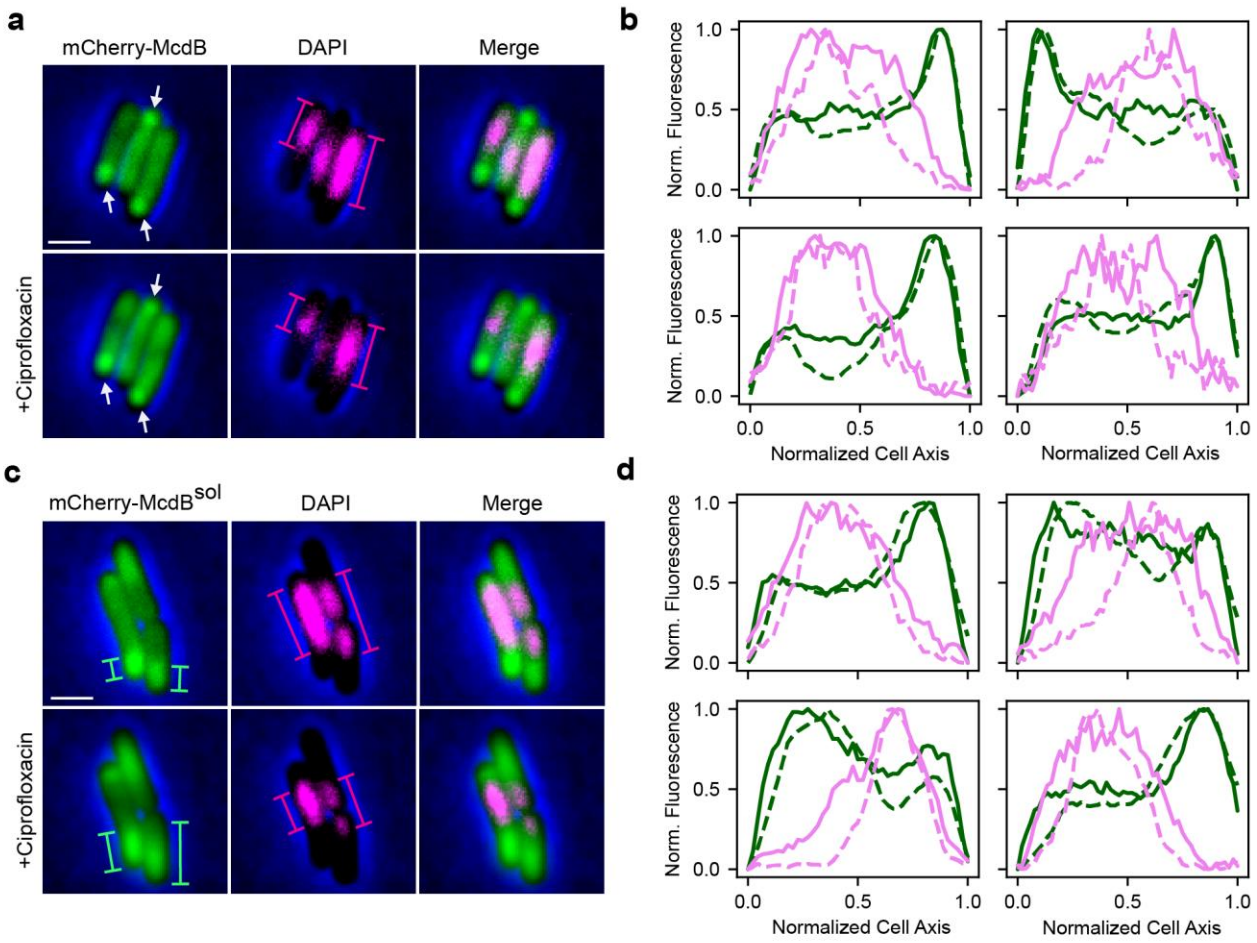

Figure S6. mCherry-McdB^{sol} condenses by repulsive interactions with the nucleoid. a. Representative images of mCherry-McdB foci in *E. coli* at 4 h post-induction (top). Cells were treated with ciprofloxacin (50 mM) for nucleoid compaction and stained with DAPI (2 µM) for nucleoid visualization (bottom). mCherry channel (green), DAPI (magenta), and merged images overlaid with Phase contrast (blue) are shown. White arrows highlight the mCherry-McdB foci that remained after nucleoid compaction. Magenta bars highlight the size of the nucleoid before and after ciprofloxacin treatment. **b.** Normalized profile lines along the normalized lengths of the long axes of four representative cells (*n* = 4) from the population shown in (a). Green and magenta lines represent the mCherry and DAPI signals, respectively. Solid lines and dashed lines represent the signal before and after ciprofloxacin treatment, respectively. **c.** Same as in (a) for mCherry-McdB^{sol} cells. Green bars indicate the length of the mCherry-McdB^{sol} foci before and after ciprofloxacin treatment. **d.** Same as in (b) for cells from the population shown in (c). Images are representative of three biological replicates. Scale bar: 1 µm.

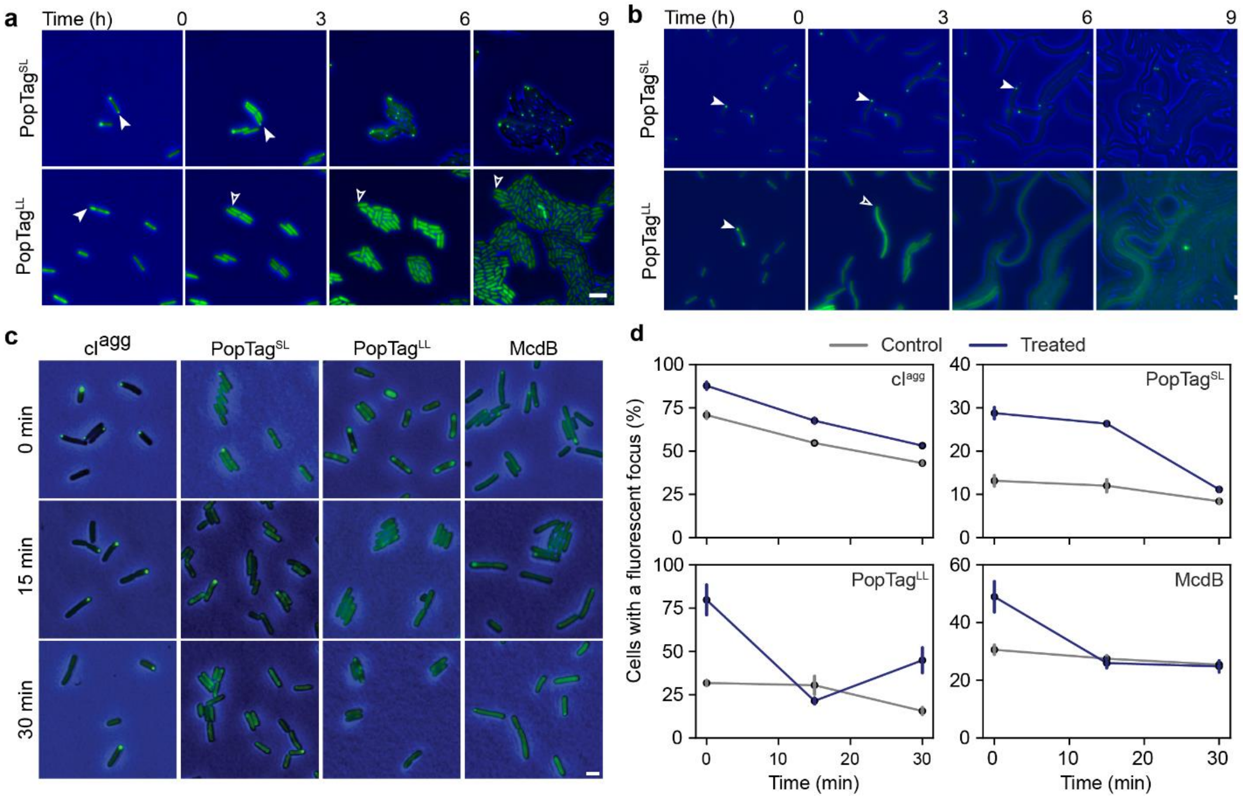

Figure S7. Condensate reversibility through cell growth, division, and osmolarity shift. a. Generational dilution dissolves phase-separated foci. The phase contrast channel (blue) and the mCherry channel (green) are merged for a-c. White arrows demarcate the same cellular location of the same focus over time. Blank arrows demarcate the cellular position now absent of a focus. Images are representative of four biological replicates. Scale bars: 2 µm. **b.** Cell elongation dissolves phase-separated foci. As in (a), except cells were treated with 10 µg/ml cephalexin to block cell division prior to spotting on agar pads. **c.** Cells osmotically shocked with 300 mM NaCl. The first row of panels shows cells treated cells pre-wash; 15 and 30 min are cells post-wash. Images are representative of three biological replicates. **d.** Percent of cells with a fluorescent focus for osmotically shocked cells shown in (c) and for untreated control cells from three biological replicates. Bars indicate the standard deviation across the replicates. *N* > 50 cells for each protein at each time point per replicate.

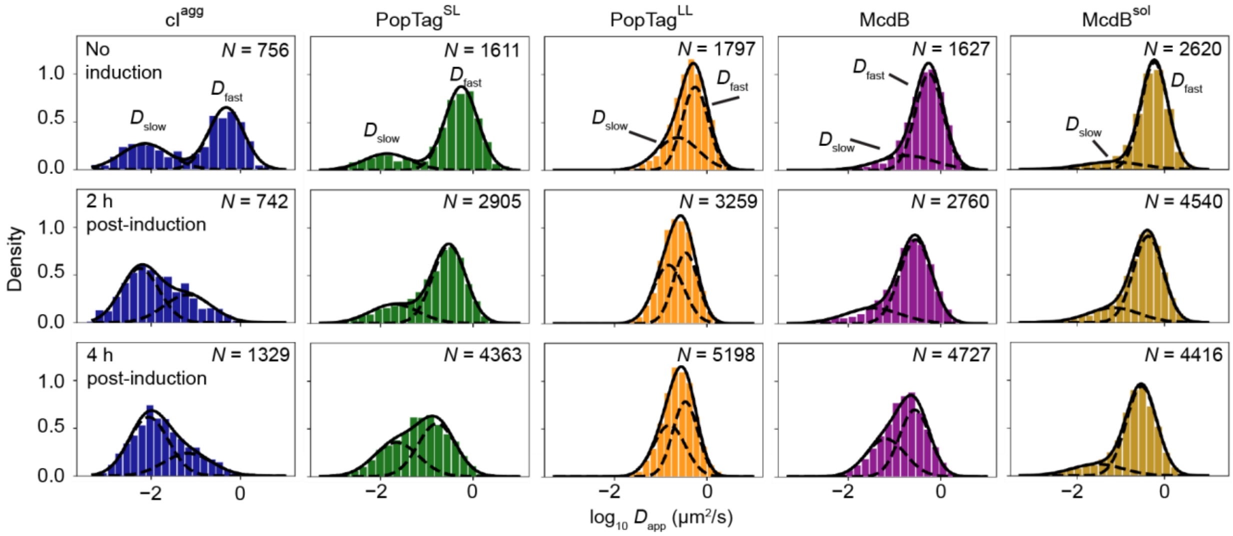

Figure S8. Diffusion coefficients of PAmCherry fusion proteins. Normalized log apparent diffusion coefficient histograms and two-component Gaussian mixture fit. The solid black line corresponds to the two-component Gaussian fit. The dashed black lines represent the Gaussian fit of each component which we refer to as $D_{app,slow}$ and $D_{app,fast}$. *N* indicates the number of trajectories analyzed for each protein per condition.

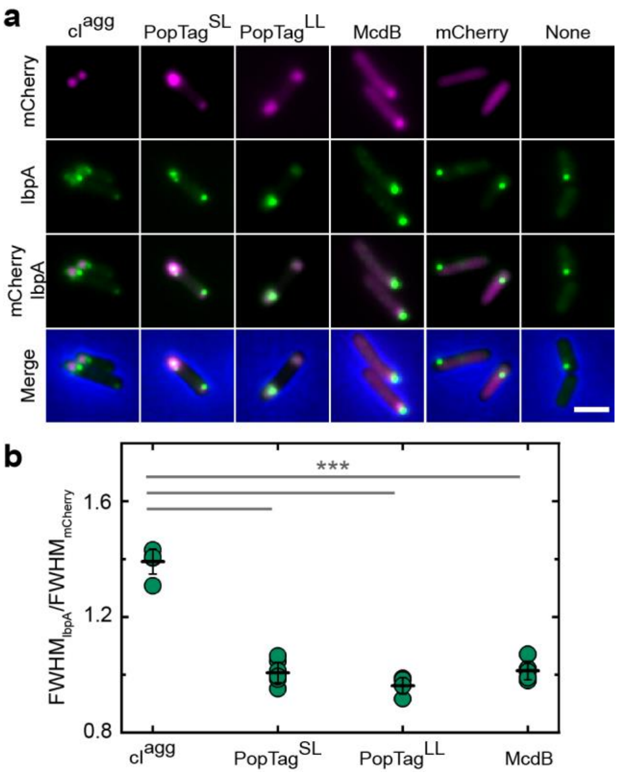

Figure S9. The nature of IbpA chaperone association with foci informs their material state *in vivo*. Wide-field fluorescence images of mCherry fusion foci (magenta) and IbpA-msfGFP foci (green). Phase Contrast is blue in the merge. Images are representative of three biological replicates. Scale bar: 2 µm. **b.** Quantification of the association of the foci of mCherry fusions and IbpA-msfGFP. Data points correspond to the ratio of the mean full-width at half maximum (FWHM) for the two channels ($FWHM_{IbpA}/FWHM_{mCherry}$) for the sum projections of technical replicates (cI^{agg}: 5, PopTag^{SL}: 7, PopTag^{LL}: 4, McdB: 5) over three independent experiments. n > 30 foci for each protein per technical replicate. Black lines represent the mean ratio and the error bars represent the standard deviation. ***$p < 0.001$ by Welch's *t*-test.

Supplementary Table 1. Focus dissolution tracking analysis

Figure	Protein	Number of foci tracked	Number of foci that dissolved	% of foci that dissolved	Lifespan of dissolved foci (h)
4a-b	cI^{agg}	93	0	0	na
4a-b	$PopTag^{SL}$	148	111	75	1.6 ± 2
4a-b	$PopTag^{LL}$	64	64	100	0.3 ± 0.2
4a-b	McdB	87	84	96.6	1.4 ± 1
4d-e	cI^{agg}	102	0	0	na
4d-e	$PopTag^{SL}$	119	95	75	4.3 ± 2.3
4d-e	$PopTag^{LL}$	35	35	100	0.3 ± 0.1

Supplementary Table 2. Apparent diffusion coefficients and weight fraction resulting from two-state Gaussian mixture fitting (Fig. 5c-e and S8).

Protein	Sample induction (100 µM)	$D_{app,slow}$ (µm^2/s)	π_{slow}	$D_{app,fast}$ (µm^2/s)	π_{fast}
cI^{agg}	None	0.007 ± 0.001	0.38 ± 0.02	0.467 ± 0.024	0.62 ± 0.02
	2 h	0.006 ± 0.001	0.62 ± 0.03	0.062 ± 0.006	0.38 ± 0.03
	4 h	0.008 ± 0.001	0.65 ± 0.03	0.074 ± 0.006	0.35 ± 0.03
$PopTag^{SL}$	None	0.013 ± 0.001	0.20 ± 0.01	0.572 ± 0.016	0.80 ± 0.01
	2 h	0.024 ± 0.002	0.28 ± 0.02	0.314 ± 0.010	0.72 ± 0.02
	4 h	0.023 ± 0.001	0.43 ± 0.02	0.165 ± 0.006	0.57 ± 0.02
$PopTag^{LL}$	None	0.234 ± 0.021	0.37 ± 0.04	0.559 ± 0.026	0.63 ± 0.04
	2 h	0.143 ± 0.006	0.47 ± 0.03	0.347 ± 0.008	0.53 ± 0.03
	4 h	0.158 ± 0.004	0.46 ± 0.03	0.347 ± 0.012	0.54 ± 0.03
McdB	None	0.153 ± 0.026	0.23 ± 0.05	0.576 ± 0.022	0.77 ± 0.05
	2 h	0.038 ± 0.005	0.23 ± 0.02	0.293 ± 0.006	0.77 ± 0.02
	4 h	0.065 ± 0.003	0.40 ± 0.02	0.268 ± 0.008	0.60 ± 0.02
$McdB^{sol}$	None	0.063 ± 0.017	0.14 ± 0.01	0.581 ± 0.010	0.87 ± 0.01
	2 h	0.068 ± 0.008	0.23 ± 0.01	0.437 ± 0.009	0.77 ± 0.01
	4 h	0.030 ± 0.003	0.19 ± 0.01	0.297 ± 0.003	0.81 ± 0.01

Supplementary Table 3. Strain and plasmids used in this study

Strains & Plasmids	Description	Source
**Strains**		
BL21 AE		
BL21 AI		
MG1655 Δ*lacY*		
MG1655 Δ*lacY ibpA-msfgfp*	MG1655 encoding a C-terminal fusion of IbpA to msfGFP	This study
**Plasmids**		
	pET-*mNeonGreen-mcdB*	This study
	pET-His_6-*mNeonGreen-mcdB*	This study
pYH79	pET-His_6-*mCherry-mcdB*	This study
pTrc99A-*mCherry-cI78*^{EP8}	pTrc99A expression vector allowing the inducible expression (P_{trc} promoter) of a N-terminal fusion of cI78^{EP8} (a mutagenized fragment of the lambda prophage repressor protein) to the mCherry fluorescent protein	[28]
pCA3	pTrc99A-PAmCherry- *cI78*^{EP8}	This study
pYH70	pTrc99A-*PAmCherry-mcdB*	This study
pYH71	pTrc99A-*mCherry-mcdB*	This study
pYH74	pTrc99A-*mNeonGreen-mcdB*	This study
pYH75	Ptrc99A-*mCherry-L6-PopTag*	This study
pYH76	Ptrc99A-*PAmCherry-L6-PopTag*	This study
pYH77	Ptrc99A-*mCherry*	This study
pYH78	Ptrc99A-*PAmCherry*	This study
pYH80	Ptrc99A-*mCherry-L78-PopTag*	This study
pYH88	trc99A-*PAmCherry-L78-PopTag*	This study
pYH86	Ptrc-*mCherry-mcdB*^{sol}	This study
pYH89	Ptrc-*PAmCherry-mcdB*^{sol}	This study

Supplementary Table 4 Primers used in this study.

Primers	Description	Note
YH1 F	CACAGGAAACAGACCATGGCAGTGAGCAAGGGCGAGGAGG	To make pTrc99A-PAmCherry-*cI78*^{EP8} from pTrc99A-*mCherry*-*cI78*^{EP8}
YH1 R	CTGCTGCCGCTGCCGGATCCCTTGTACAGCTCGTCCATGC	
YH2 F	TGCCATGGTCTGTTTCCTGTG	
YH2 R	GGATCCGGCAGCGGCAGCAG	
YH3 F	CACAGGAAACAGACCATGGCAGTGAGCAAGGGCGAGGAGG	To make pYH70 from pYH71
YH3 R	CATGCTGCCGCTGCCGGATCCCTTGTACAGCTCGTCCATGC	
YH4 F	TGCCATGGTCTGTTTCCTG	
YH4 R	GGATCCGGCAGCGGCAGCATG	
YH5 F	AGGGATCCGGCAGCGGCAGCATGACTGACGCATTCGACCG	To make pYH71 from pTrc99A-*mCherry*-*cI78*^{EP8}
YH5 R	CTTGCATGCCTGCAGGTCGACTCACCAACCGTGCAGCTTG	
YH6 F	GCTGCCGCTGCCGGATCCCT	
YH6 R	GTCGACCTGCAGGCATGCAAG	
YH7 F	CACAGGAAACAGACCATGGCAGTGTCGAAAGGAGAAG	To make pYH74 from pYH71
YH7 R	CATGCTGCCGCTGCCGGATCCCTTGTATAATTCGTCCATC	
YH8 F	AGGGATCCGGCAGCGGCAGCATGGTCGCGGAACAACTTG	To make pYH75 from pYH74
YH8 R	GCATGCCTGCAGGTCGACTCATTAAGCTCCGCGACCACG	
YH9	GGATCCGGCAGCGGCAGCAT	To make pYH76 from pYH75
YH10	ATGCTGCCGCTGCCGGATCCCTTGTACAGCTCGTCCATGC	
YH11	GTCGACCTGCAGGCATGCA	To make pYH77 from pYH78
YH12	TTGCATGCCTGCAGGTCGACTCACTTGTACAGCTCGTCC	

Supplementary Table 5. Reagents used in this study.

Reagent	Vendor	Catalog #	Use
4-12% Bis-Tris NuPAGE gel	Thermo Fisher Scientific	NP0323BOX	SDS-PAGE
Bradford assay kit	Bio-Rad Laboratories	5000006	To measure protein content
Carbenicillin	Thermo Fisher Scientific	MT46100RG	To select for the plasmid in culture
DAPI	Thermo Fisher Scientific	D1306	To label the nucleoid
Glucose	Thermo Fisher Scientific	15023021	As a carbon source and to inhibit protein expression from the P_{trc} promoter
Glycerol	Thermo Fisher Scientific	G334	As a carbon source for *E. coli* MG1665
Goat anti-rabbit IgG Secondary Antibody IRDye 800 CW	LI-COR	926-32210	For western blots
IPTG	Thermo Fisher Scientific	501126935	To induce protein expression
L-arabinose	Thermo Fisher Scientific	AC365181000	To induce protein expression
Mini-size polyvinylidene difluoride membrane	Bio-Rad	1704156	For protein transfer during western blots
Cephalexin	Thermo Fisher Scientific	AAJ6317206	To inhibit cell division
A22	Millipore Sigma	SML0471	As the MreB inhibitor, which converts rod-shaped into round cells
Casamino acids	Millipore Sigma	65072-00-6	For AB medium
Thiamine	Millipore Sigma	T4625-5G	For AB medium
Uracil	Millipore Sigma	66-22-8	For AB medium
UltraPure agarose	Invitrogen	16500100	For agar pads
Ciprofloxacin	Thermo Fisher Scientific	AAJ6131714	To compact the nucleoid

Supplementary Table 6. Parameters used to detect fluorescent foci for classification

Protein	*norm_thresh*	*eccent_thresh*
cI^{agg}	0.55	0.85
$PopTag^{SL}$	0.65	0.85
$PopTag^{LL}$	0.75	0.85
McdB	0.8	0.85
$McdB^{sol}$	0.75	0.85
mCherry	0.75	0.85
