# Supplementary material for: An experimental framework to assess biomolecular condensates in bacteria
Source: Nat Commun. 2024 Apr 15;15:3222. doi: 10.1038/s41467-024-47330-4 (PMC11018776; doi:10.1038/s41467-024-47330-4)
Supplement: Supplementary file 10 — Reporting Summary [file 41467_2024_47330_MOESM10_ESM.pdf]

nature portfolio

Corresponding author(s): Julie S. Biteen, Anthony G. Vecchiarelli

Last updated by author(s): Nov 29, 2023

Reporting Summary

Nature Portfolio wishes to improve the reproducibility of the work that we publish. This form provides structure for consistency and transparency in reporting. For further information on Nature Portfolio policies, see our Editorial Policies and the Editorial Policy Checklist.

Statistics

For all statistical analyses, confirm that the following items are present in the figure legend, table legend, main text, or Methods section.

n/a	Confirmed	
☐	☒	The exact sample size (*n*) for each experimental group/condition, given as a discrete number and unit of measurement
☐	☒	A statement on whether measurements were taken from distinct samples or whether the same sample was measured repeatedly
☐	☒	The statistical test(s) used AND whether they are one- or two-sided<br>*Only common tests should be described solely by name; describe more complex techniques in the Methods section.*
☒	☐	A description of all covariates tested
☐	☒	A description of any assumptions or corrections, such as tests of normality and adjustment for multiple comparisons
☐	☒	A full description of the statistical parameters including central tendency (e.g. means) or other basic estimates (e.g. regression coefficient) AND variation (e.g. standard deviation) or associated estimates of uncertainty (e.g. confidence intervals)
☐	☒	For null hypothesis testing, the test statistic (e.g. *F*, *t*, *r*) with confidence intervals, effect sizes, degrees of freedom and *P* value noted<br>*Give P values as exact values whenever suitable.*
☒	☐	For Bayesian analysis, information on the choice of priors and Markov chain Monte Carlo settings
☒	☐	For hierarchical and complex designs, identification of the appropriate level for tests and full reporting of outcomes
☒	☐	Estimates of effect sizes (e.g. Cohen's *d*, Pearson's *r*), indicating how they were calculated

Our web collection on statistics for biologists contains articles on many of the points above.

Software and code

Policy information about availability of computer code

Data collection	NIS Elements AR 5.02.01 software was used for image acquisition. Super-resolution image reconstruction was perform using the Nikon Elements SIM module. Single molecule data was collected with NIS Elements D 3.1 software.
Data analysis	Data processing and analysis scripts for this study were written in MATLAB and Python. All image analysis scripts, the SMALL-LABS and NOBIAS algorithm packages are available on GitHub (http://github.com/BiteenMatlab).<br>mCherry cleavage graph was made in GraphPad Prism Version 9 (GraphPad Software, San Diego, CA, www.graphpad.com).

For manuscripts utilizing custom algorithms or software that are central to the research but not yet described in published literature, software must be made available to editors and reviewers. We strongly encourage code deposition in a community repository (e.g. GitHub). See the Nature Portfolio guidelines for submitting code & software for further information.

Data

Policy information about availability of data

All manuscripts must include a data availability statement. This statement should provide the following information, where applicable:

- Accession codes, unique identifiers, or web links for publicly available datasets
- A description of any restrictions on data availability
- For clinical datasets or third party data, please ensure that the statement adheres to our policy

All data generated or analyzed during this study are included in this published article and its supplementary information files. Source data are also provided with this

paper.

Research involving human participants, their data, or biological material

Policy information about studies with human participants or human data. See also policy information about sex, gender (identity/presentation), and sexual orientation and race, ethnicity and racism.

Reporting on sex and gender	NA
Reporting on race, ethnicity, or other socially relevant groupings	NA
Population characteristics	NA
Recruitment	NA
Ethics oversight	NA

Note that full information on the approval of the study protocol must also be provided in the manuscript.

Field-specific reporting

Please select the one below that is the best fit for your research. If you are not sure, read the appropriate sections before making your selection.

[x] Life sciences [ ] Behavioural & social sciences [ ] Ecological, evolutionary & environmental sciences

For a reference copy of the document with all sections, see nature.com/documents/nr-reporting-summary-flat.pdf

Life sciences study design

All studies must disclose on these points even when the disclosure is negative.

Sample size	Sample size was determined to achieve a confidence level of 95% that the real value is within 5% of the measured value. To achieve this, data sets analyze at least 200 cells for each bacterial strain except in Figure S1 d&f and Figure S6 b&d.
Data exclusions	No data was excluded from publication.
Replication	Technical replication - Imaging of strains were performed on at least three Field of Views (FOVs) and on different agarose pads.<br>Biological replicates - At least three different cultures, grown on different days were performed.
Randomization	Strains were grown of different days and imaged in random order. All image analysis was automated.
Blinding	During image acquisition, blinding was achieved by using the automated stage to move to at lease three random Field of Views (FOVs). All image analysis including cell segmentation and quantification of parameters for fluorescent foci were automated.

Reporting for specific materials, systems and methods

We require information from authors about some types of materials, experimental systems and methods used in many studies. Here, indicate whether each material, system or method listed is relevant to your study. If you are not sure if a list item applies to your research, read the appropriate section before selecting a response.

Materials & experimental systems

n/a	Involved in the study	
[ ]	[x]	Antibodies
[x]	[ ]	Eukaryotic cell lines
[x]	[ ]	Palaeontology and archaeology
[x]	[ ]	Animals and other organisms
[x]	[ ]	Clinical data
[x]	[ ]	Dual use research of concern
[x]	[ ]	Plants

Methods

n/a	Involved in the study	
[x]	[ ]	ChIP-seq
[x]	[ ]	Flow cytometry
[x]	[ ]	MRI-based neuroimaging

Antibodies

Antibodies used	mCherry antibody (1:2000) was gifted by Dr. Ming Li's Lab at the University of Michigan.<br>IRDye 800CW Goat anti-Rat IgG Secondary Antibody (1:5000) (Li-cor, cat# 926-32219, lot# C50108-01)

Validation	The mCherry antibody was validated with a range of purified mCherry proteins (0 - 1 ug/mL). The mCherry antibody was also validated using yeast, mammalian cell, and bacterial cells extracts with overexpressed mCherry proteins.

Plants

Seed stocks	NA
Novel plant genotypes	NA
Authentication	NA
