# Supplementary material for: An experimental framework to assess biomolecular condensates in bacteria
Source: Nat Commun. 2024 Apr 15;15:3222. doi: 10.1038/s41467-024-47330-4 (PMC11018776; doi:10.1038/s41467-024-47330-4)
Supplement: Supplementary file 12 — Description of Additional Supplementary Files [file 41467_2024_47330_MOESM12_ESM.pdf]

Title: Supplementary Movie 1.
Description: Overexpressed mNG-McdB forms foci. Indicated *E. coli* BL21 strains were induced with 1 mM IPTG for expression of mNG-McdB. Time-lapse movies from 0 to 4 h post-induction were recorded. The phase contrast channel is shown in gray and the mNG channel is in green. This video is representative of three biological replicates. Scale bar: 5 µm.

Title: Supplementary Movie 2.
Description: Effects of changing cell volume on mNG-McdB focus stability. *E. coli* cell volume was increased by treating cells with the MreB inhibitor, A22 (10 µg/ml). Scale bar: 2 µm.

Title: Supplementary Movie 3.
Description: Effects of changing cell volume on mNG-cI^{agg} focus stability. *E. coli* cell volume was increased by treating cells with the MreB inhibitor, A22 (10 µg/ml). Scale bar: 2 µm.

Title: Supplementary Movie 4.
Description: Localized lysis of the cell. One cell pole was lysed using a high-intensity laser. The time-lapse video shows solubilization of the opposing focus and shift of the fluorescent signal to the opposite end. Scale bar: 1 µm.

Title: Supplementary Movie 5.
Description: mCherry-McdB^{sol} condenses by repulsive interactions with the nucleoid. mCherry-McdB (left) and mCherry-McdB^{sol} (right) foci in *E. coli* at 4 h post-induction. Cells were treated with ciprofloxacin (50 mM) for nucleoid compaction and stained with DAPI (2 µM) for nucleoid visualization (bottom). Merged images of mCherry (green), DAPI (magenta), and phase contrast (blue) channels are shown. The video is representative of three biological replicates. Scale bars: 1 µm.

Title: Supplementary Movie 6.
Description: Generational dilution dissolves condensates. Phase contrast channel (gray), mCherry channel (green), and merged images are shown. Images are representative of four biological replicates. Scale bar: 2 µm.

Title: Supplementary Movie 7.
Description: Cell elongation dissolves phase-separated condensates. Cells were treated with 10 µg/ml cephalexin to block cell division. Phase contrast (gray), mCherry channel (green), and merged images are shown. Images are representative of four biological replicates. Scale bar: 2 µm.

Title: Supplementary Movie 8.
Description: Effects of temperature shift on focus stability. Cells that formed foci were incubated at 4 °C for one hour and then transitioned to 37 °C in a stage-top incubator. Scale bar: 1 µm.
