# Supplementary figures and images for: An experimental framework to assess biomolecular condensates in bacteria
Source: Nat Commun. 2024 Apr 15;15:3222. doi: 10.1038/s41467-024-47330-4 (PMC11018776; doi:10.1038/s41467-024-47330-4)

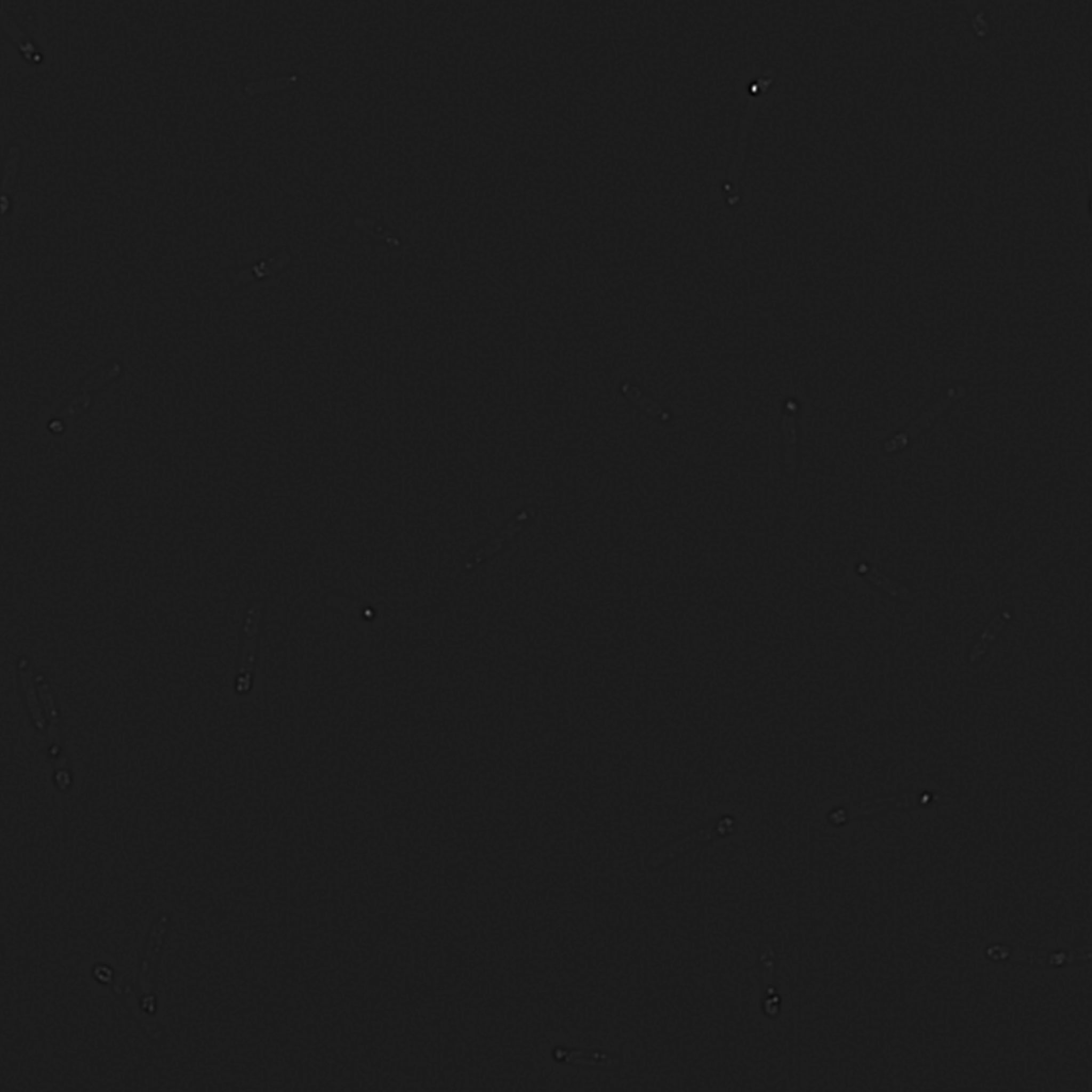

Supplement: Supplementary file 13 — Source Data [file 41467_2024_47330_MOESM13_ESM.zip › Source Data/Figure_6bc/cI_agg/cI_01.tif]

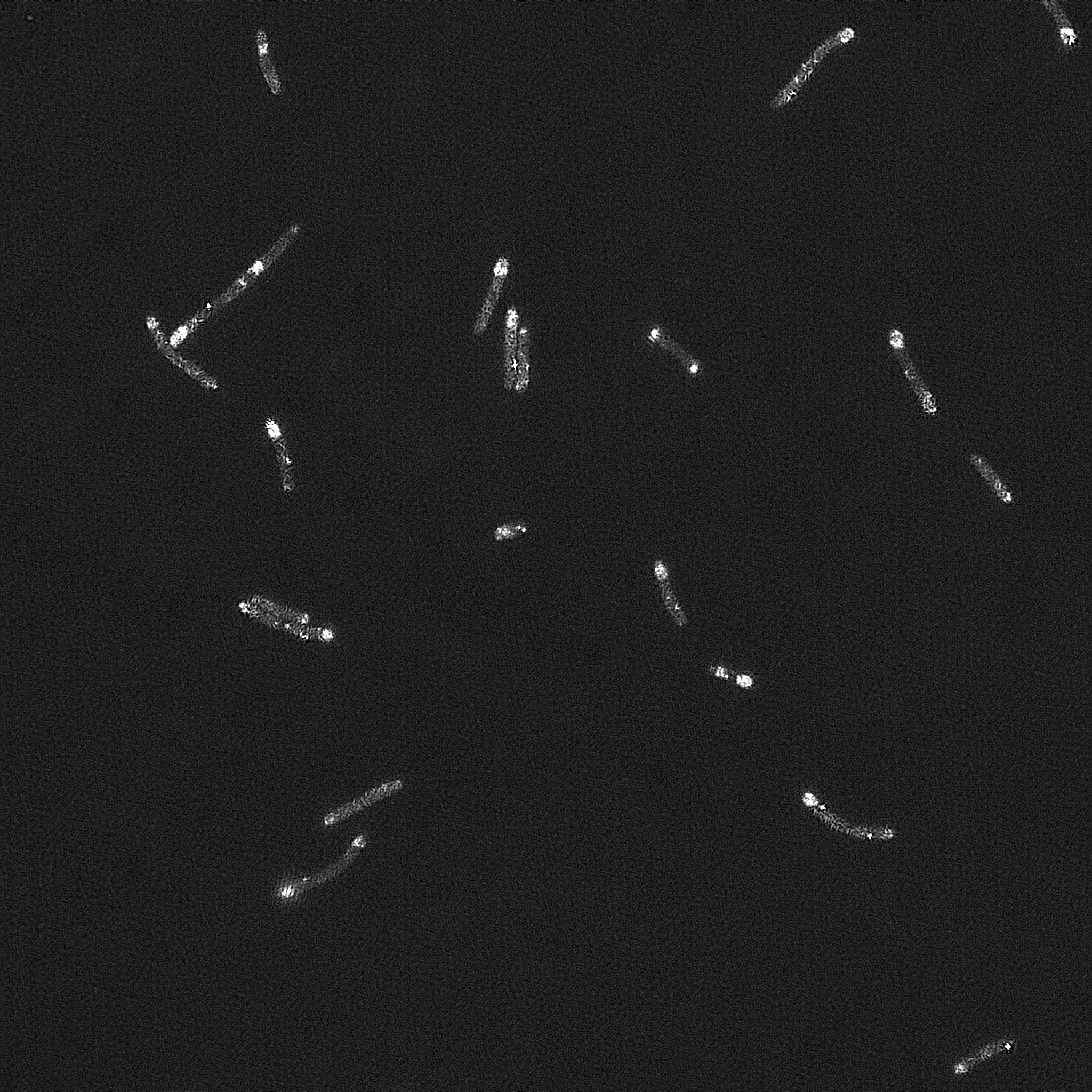

Supplement: Supplementary file 13 — Source Data [file 41467_2024_47330_MOESM13_ESM.zip › Source Data/Figure_6bc/cI_agg/cI_02.tif]

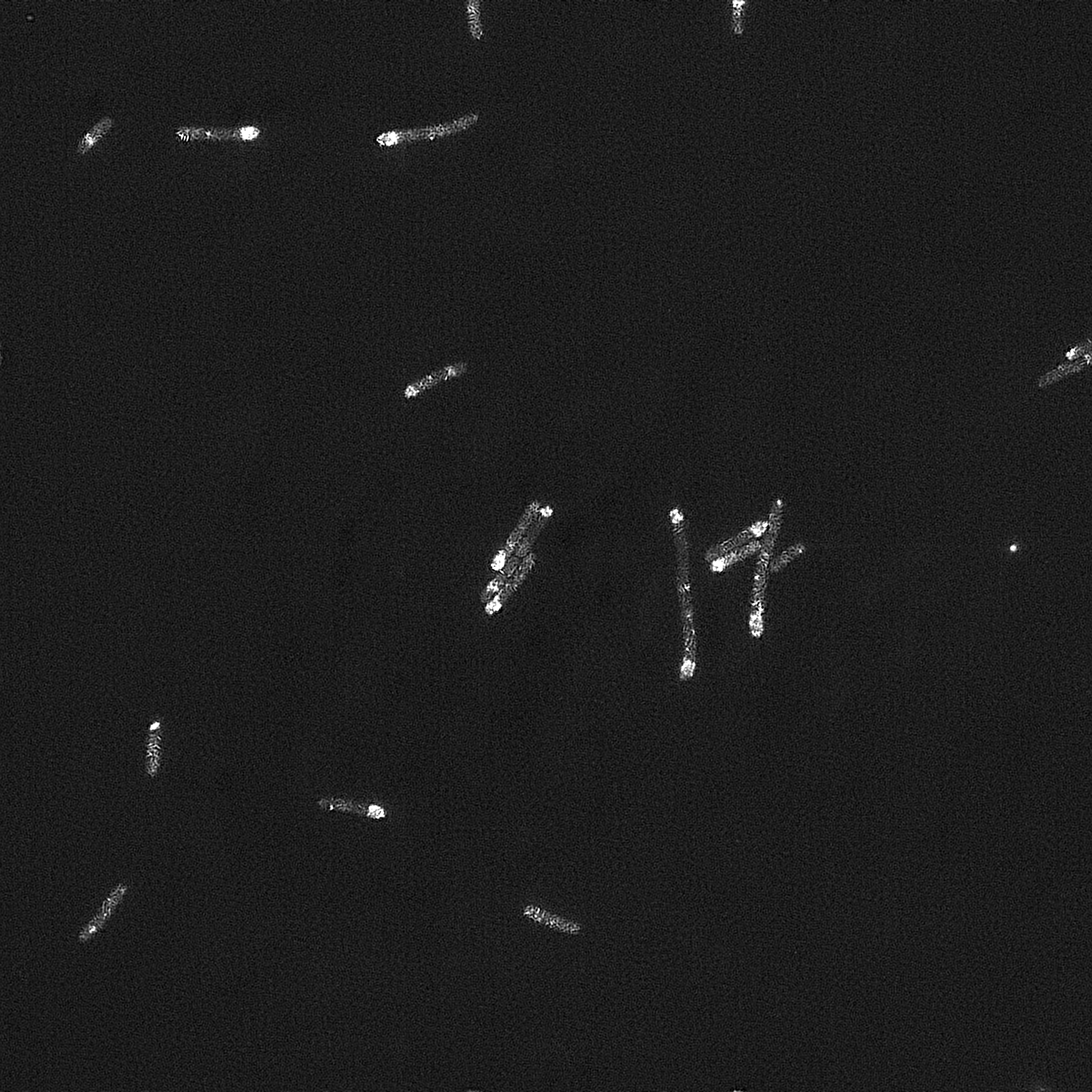

Supplement: Supplementary file 13 — Source Data [file 41467_2024_47330_MOESM13_ESM.zip › Source Data/Figure_6bc/cI_agg/cI_03.tif]

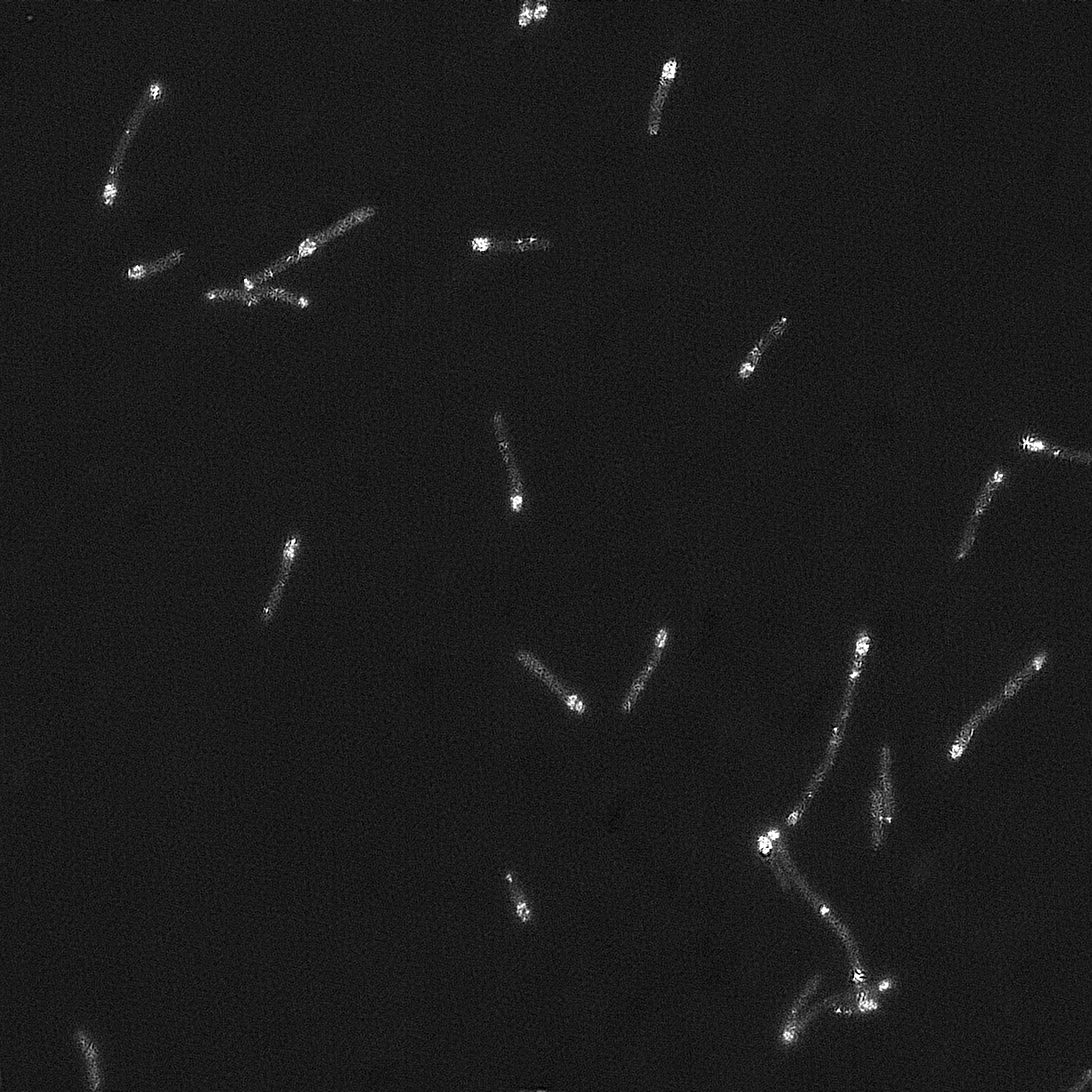

Supplement: Supplementary file 13 — Source Data [file 41467_2024_47330_MOESM13_ESM.zip › Source Data/Figure_6bc/cI_agg/cI_04.tif]

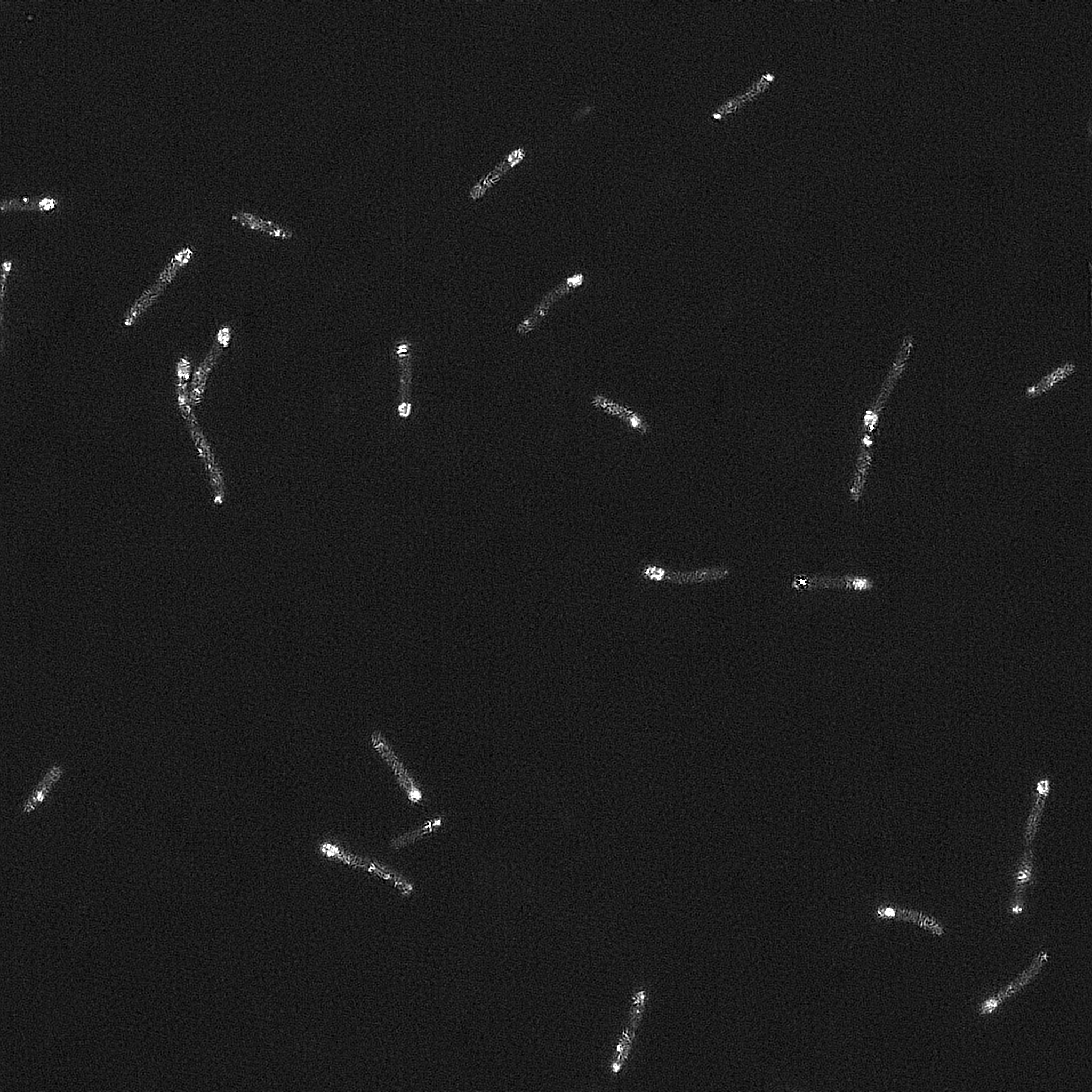

Supplement: Supplementary file 13 — Source Data [file 41467_2024_47330_MOESM13_ESM.zip › Source Data/Figure_6bc/cI_agg/cI_05.tif]

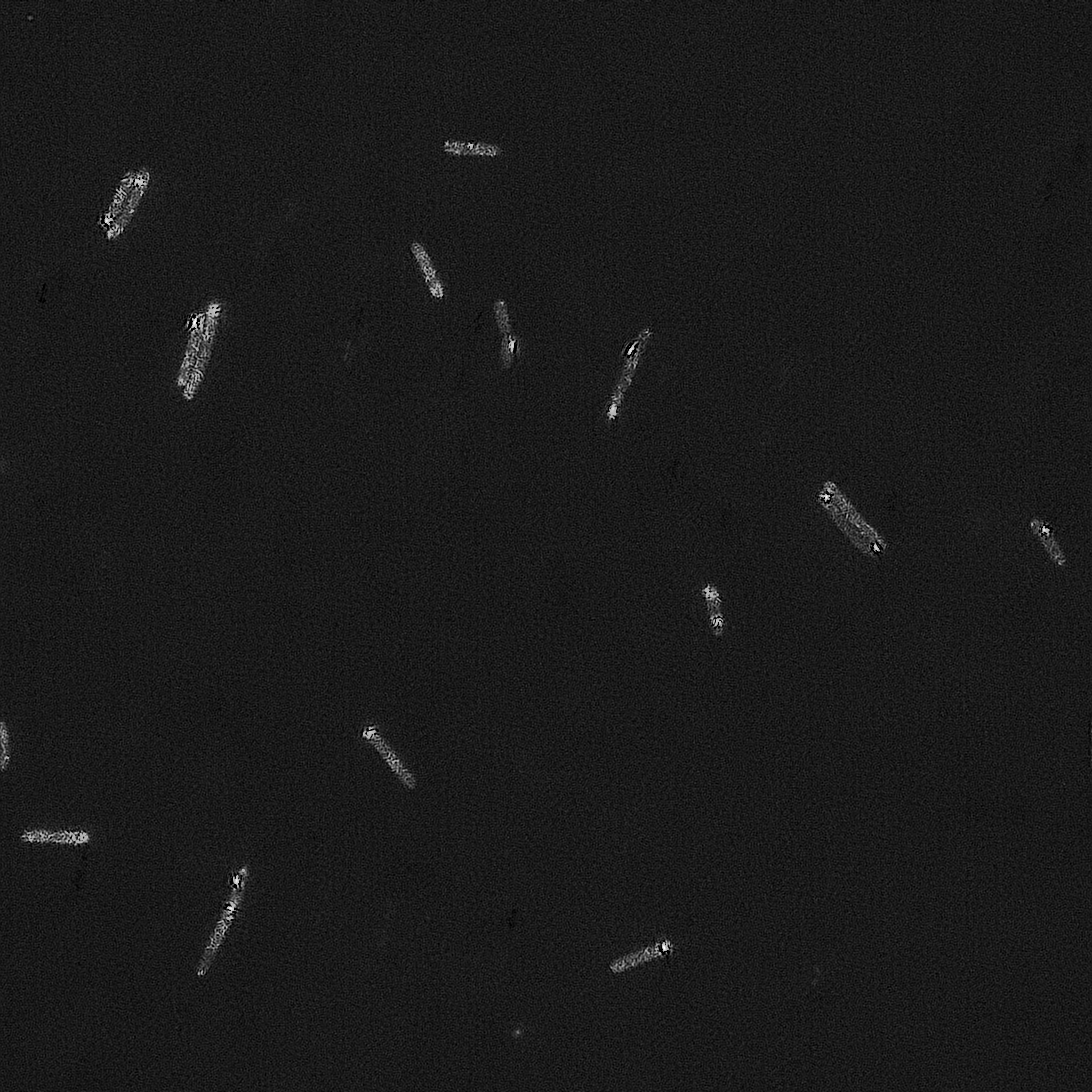

Supplement: Supplementary file 13 — Source Data [file 41467_2024_47330_MOESM13_ESM.zip › Source Data/Figure_6bc/McdB/McdB_01.tif]

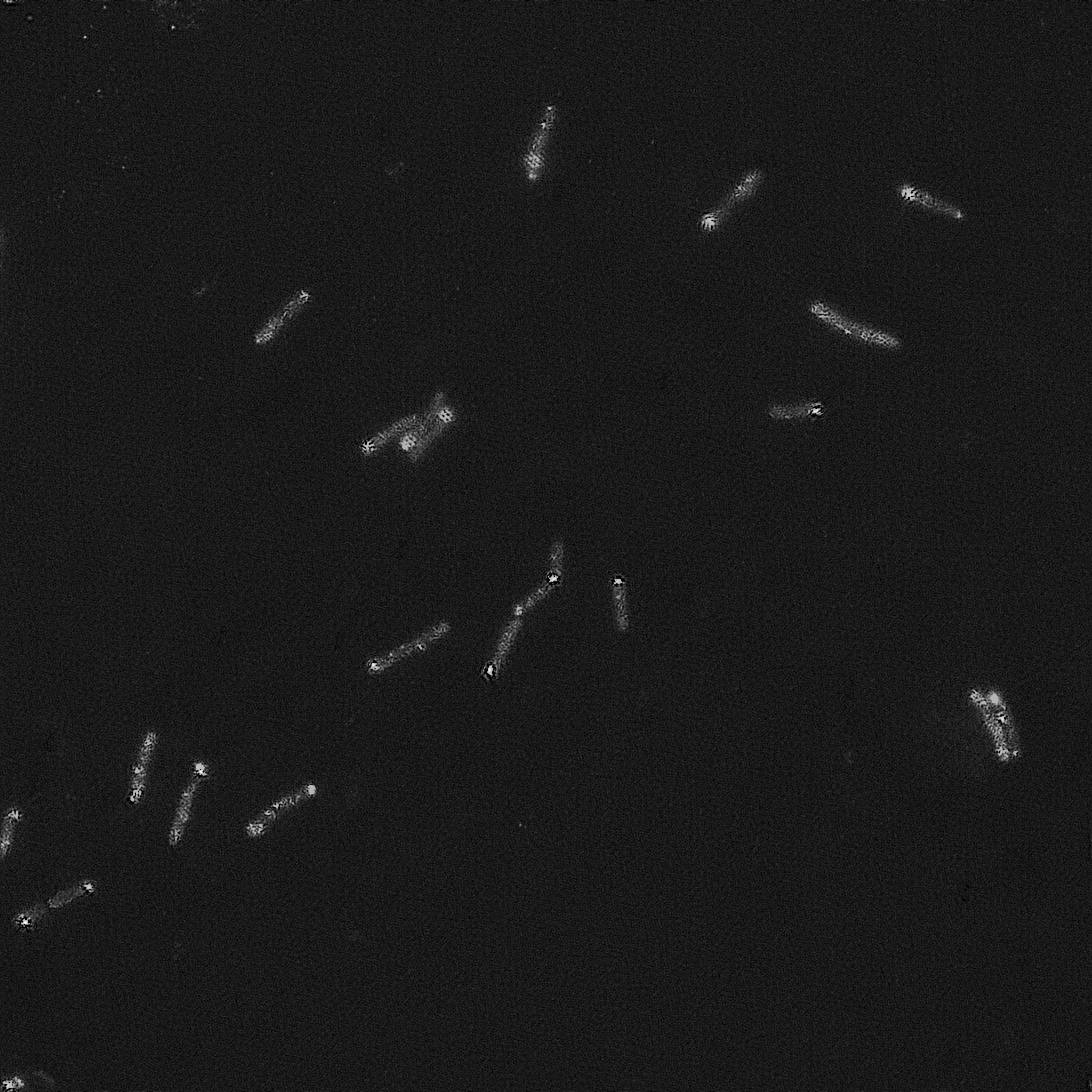

Supplement: Supplementary file 13 — Source Data [file 41467_2024_47330_MOESM13_ESM.zip › Source Data/Figure_6bc/McdB/McdB_02.tif]

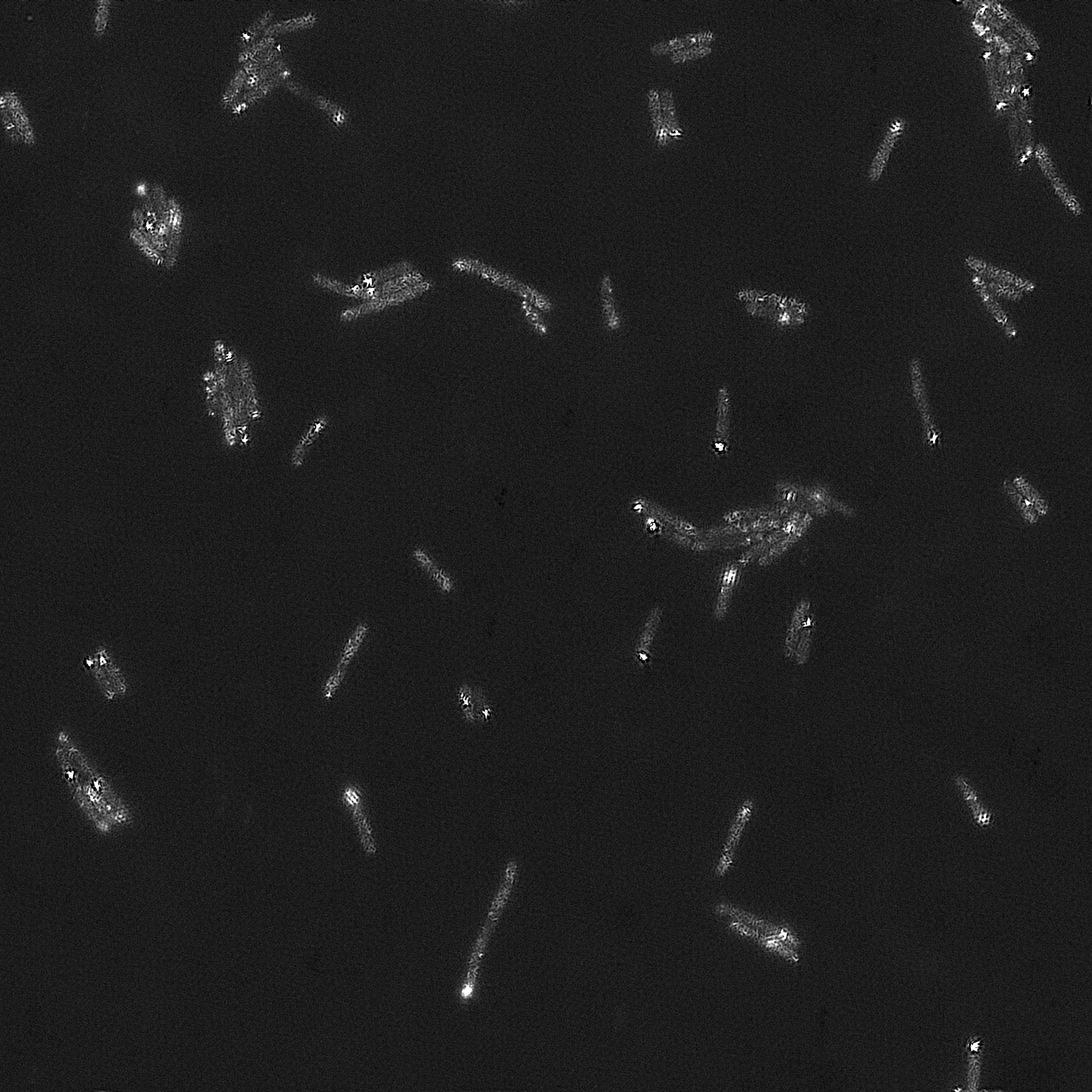

Supplement: Supplementary file 13 — Source Data [file 41467_2024_47330_MOESM13_ESM.zip › Source Data/Figure_6bc/McdB/McdB_03.tif]

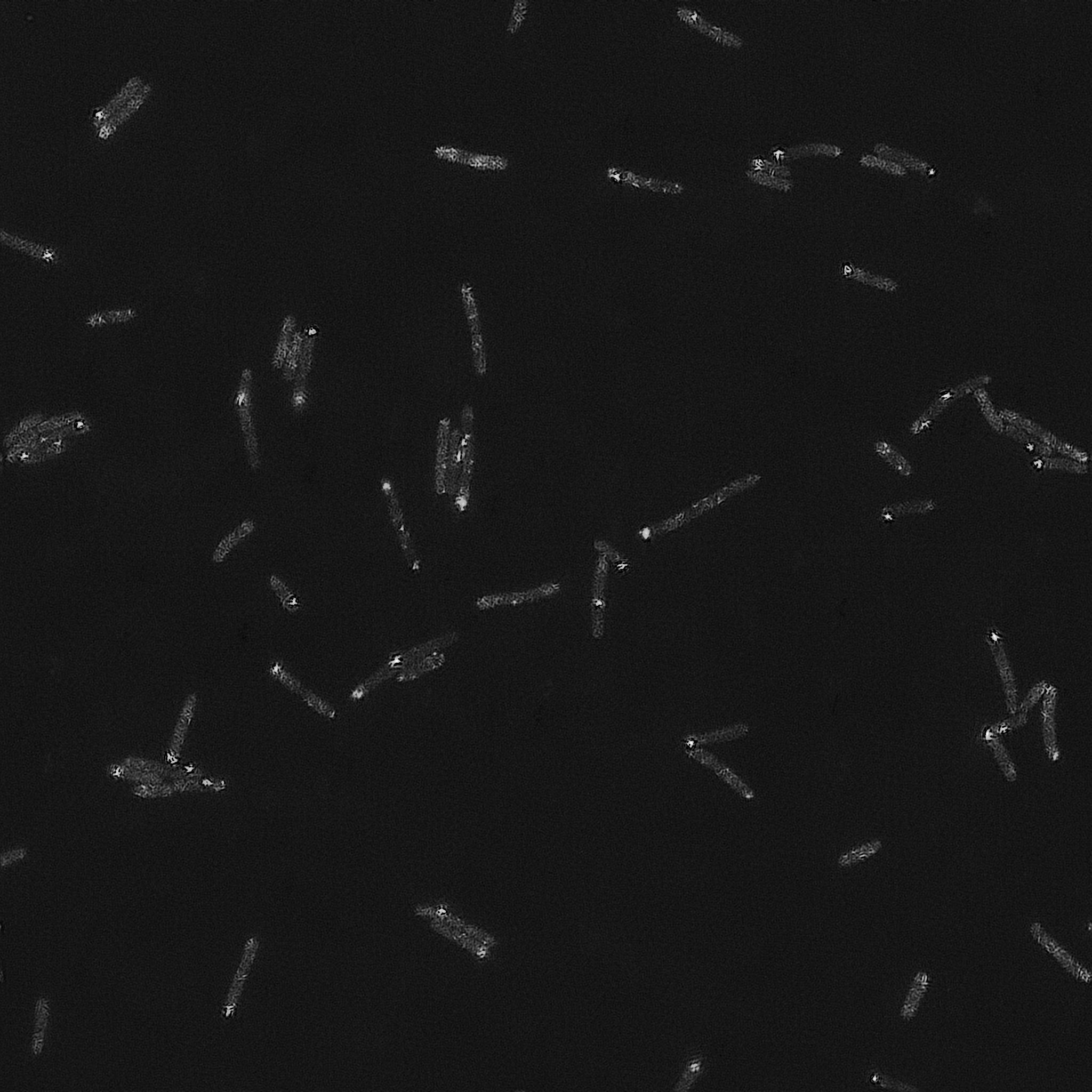

Supplement: Supplementary file 13 — Source Data [file 41467_2024_47330_MOESM13_ESM.zip › Source Data/Figure_6bc/McdB/McdB_04.tif]

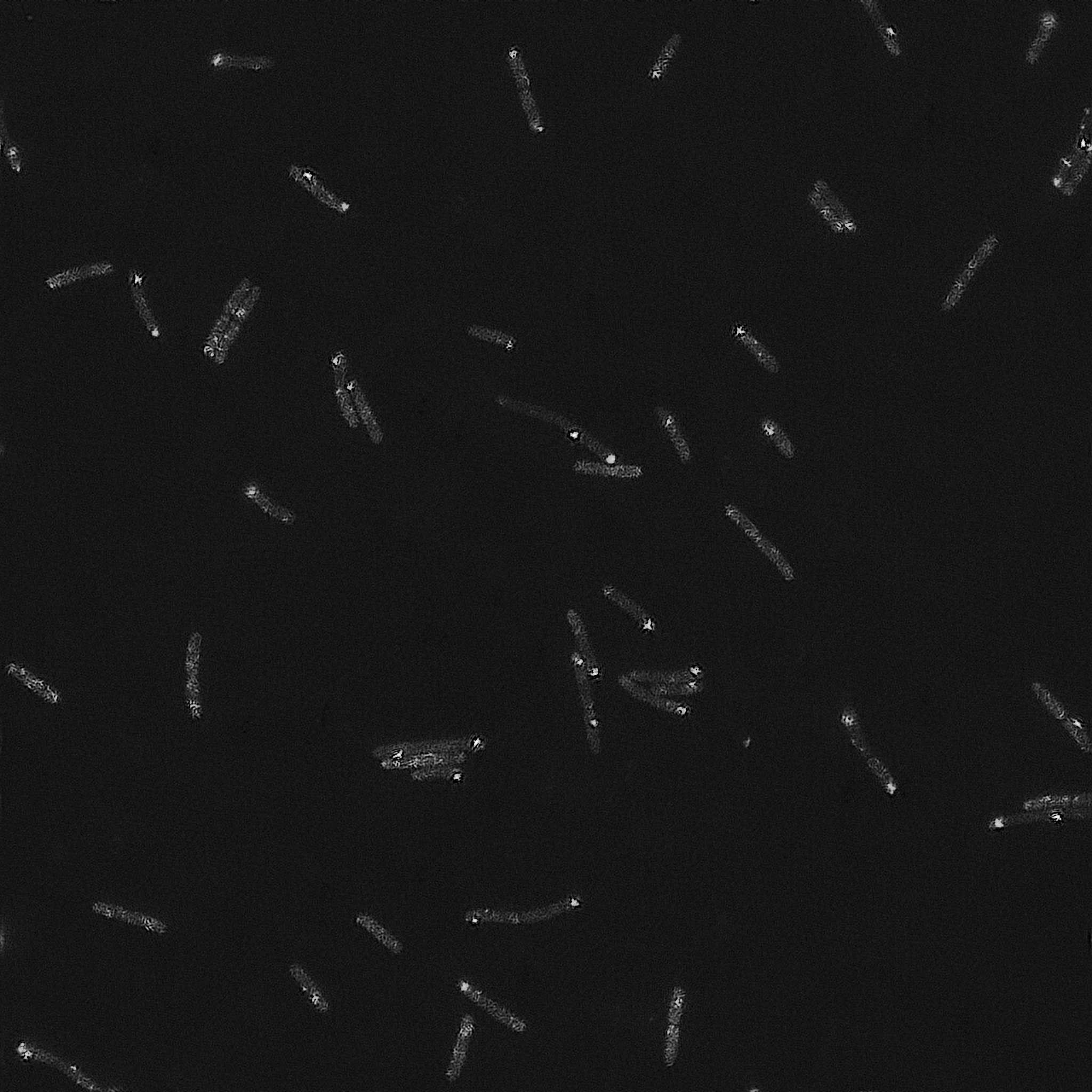

Supplement: Supplementary file 13 — Source Data [file 41467_2024_47330_MOESM13_ESM.zip › Source Data/Figure_6bc/McdB/McdB_05.tif]

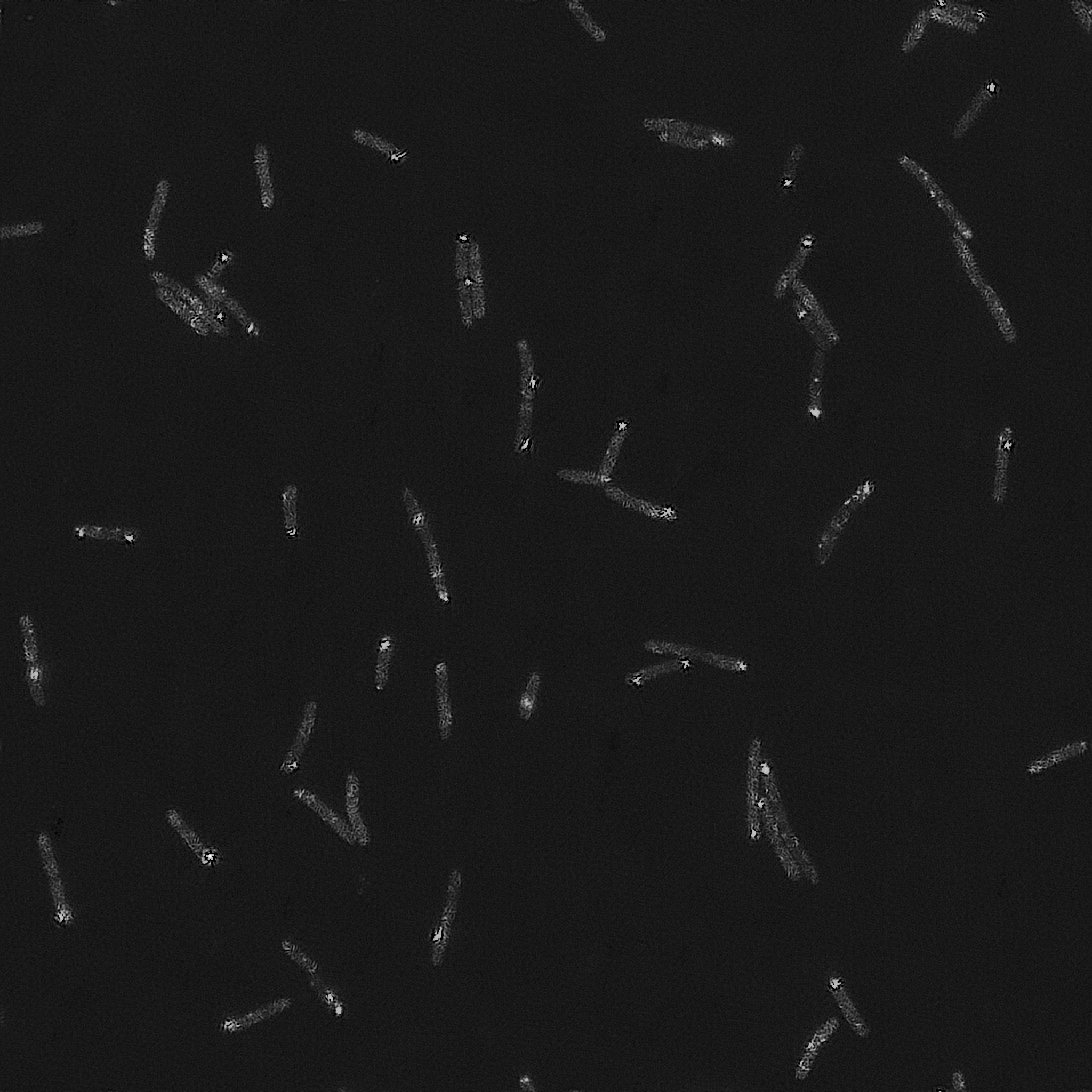

Supplement: Supplementary file 13 — Source Data [file 41467_2024_47330_MOESM13_ESM.zip › Source Data/Figure_6bc/McdB/McdB_06.tif]

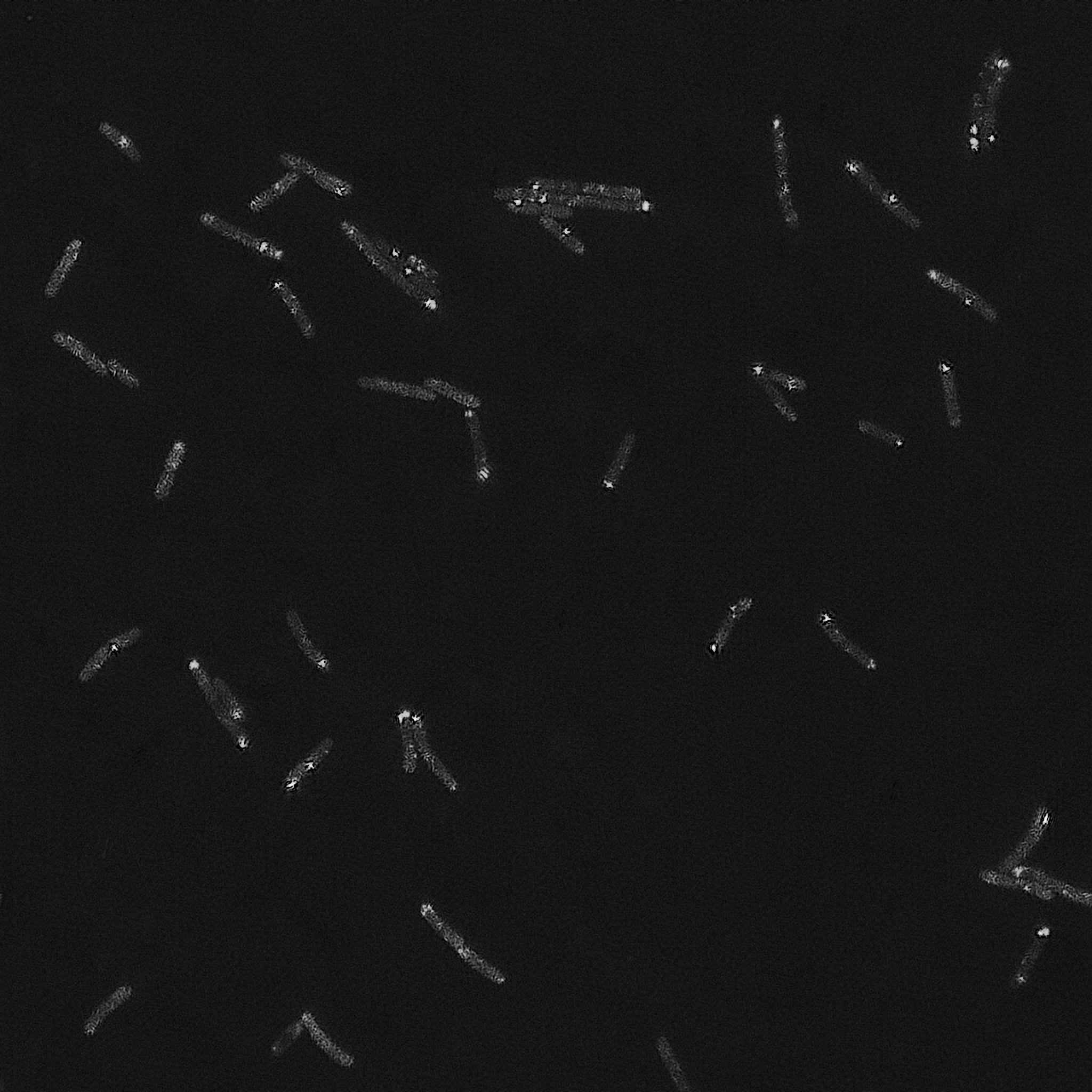

Supplement: Supplementary file 13 — Source Data [file 41467_2024_47330_MOESM13_ESM.zip › Source Data/Figure_6bc/McdB/McdB_07.tif]

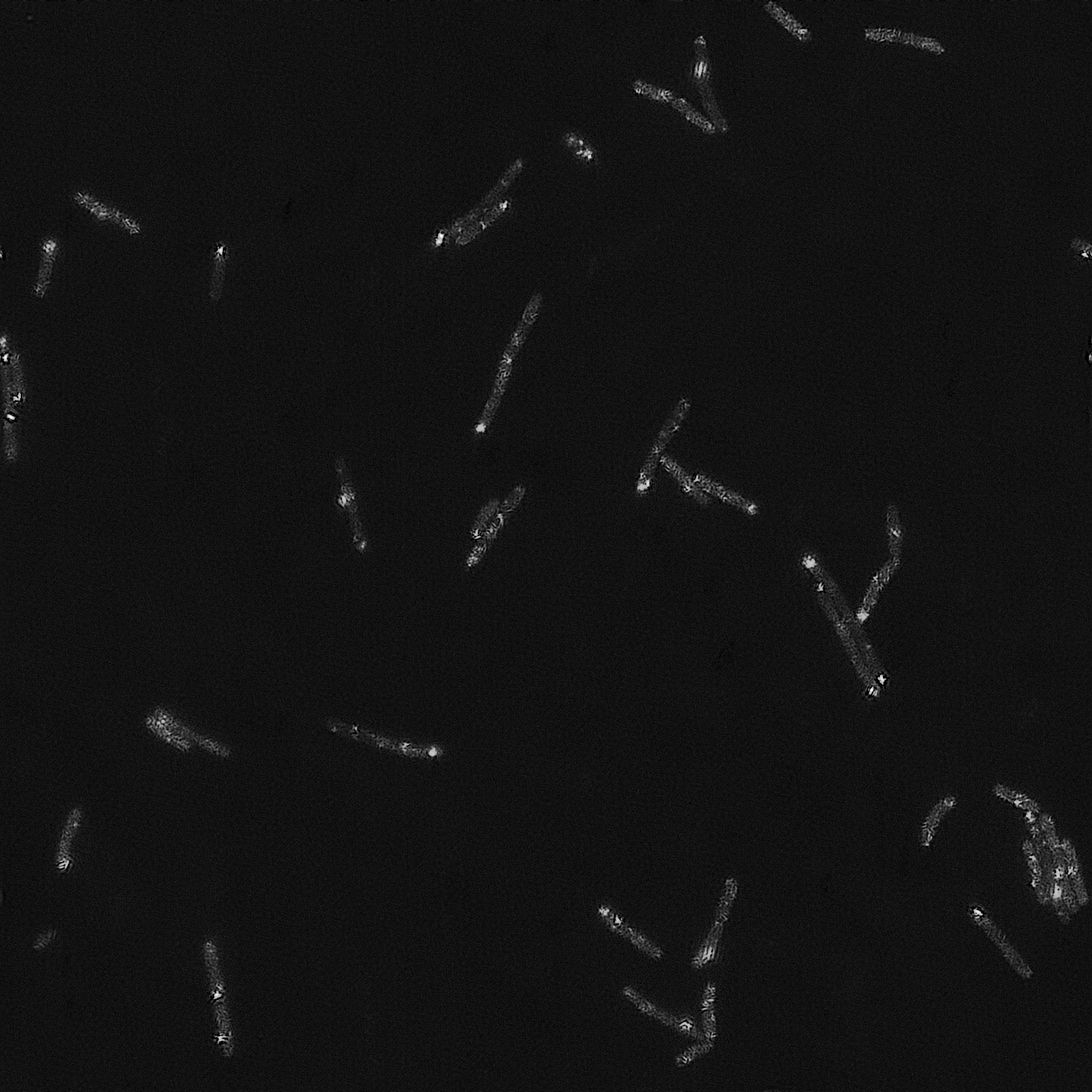

Supplement: Supplementary file 13 — Source Data [file 41467_2024_47330_MOESM13_ESM.zip › Source Data/Figure_6bc/McdB/McdB_08.tif]

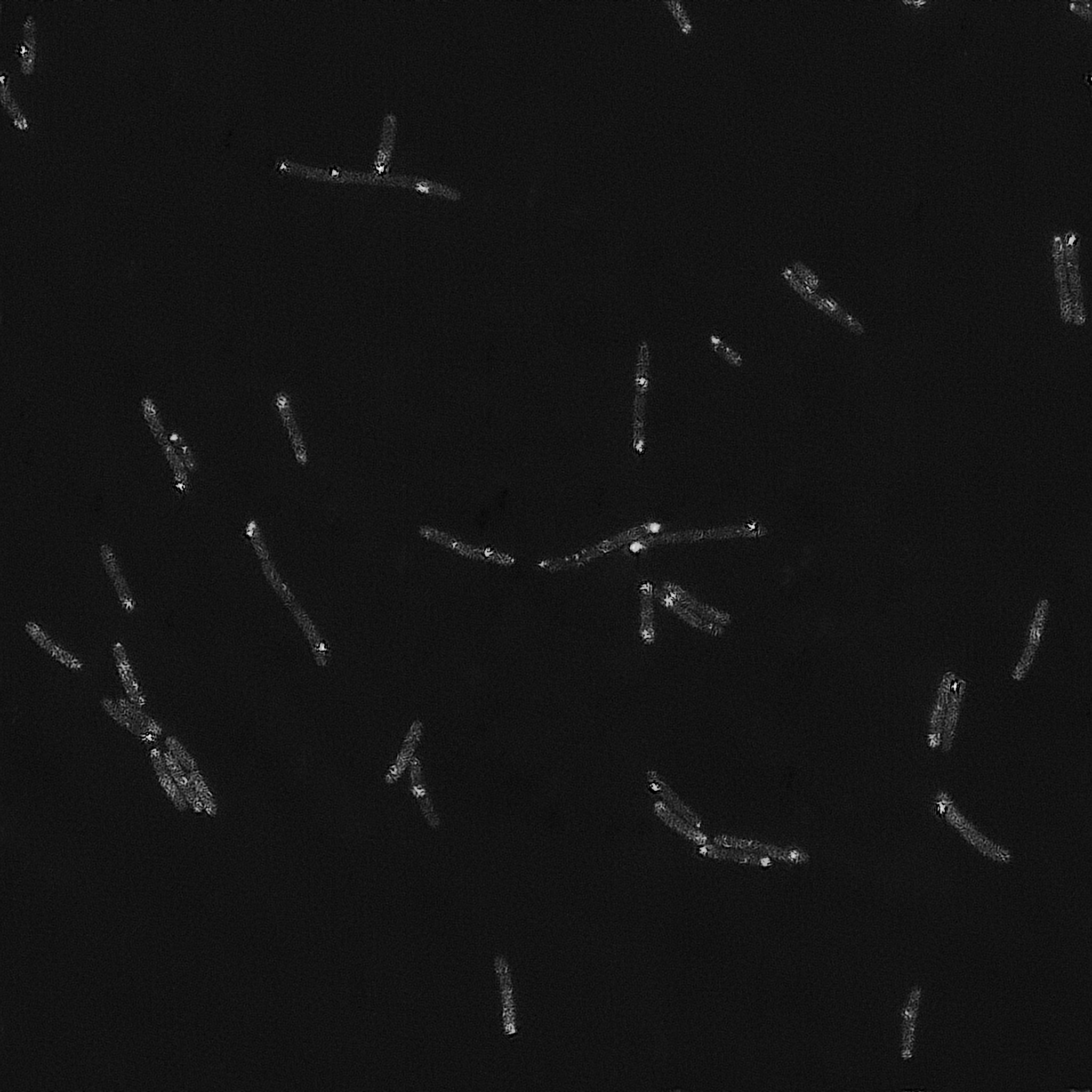

Supplement: Supplementary file 13 — Source Data [file 41467_2024_47330_MOESM13_ESM.zip › Source Data/Figure_6bc/McdB/McdB_09.tif]

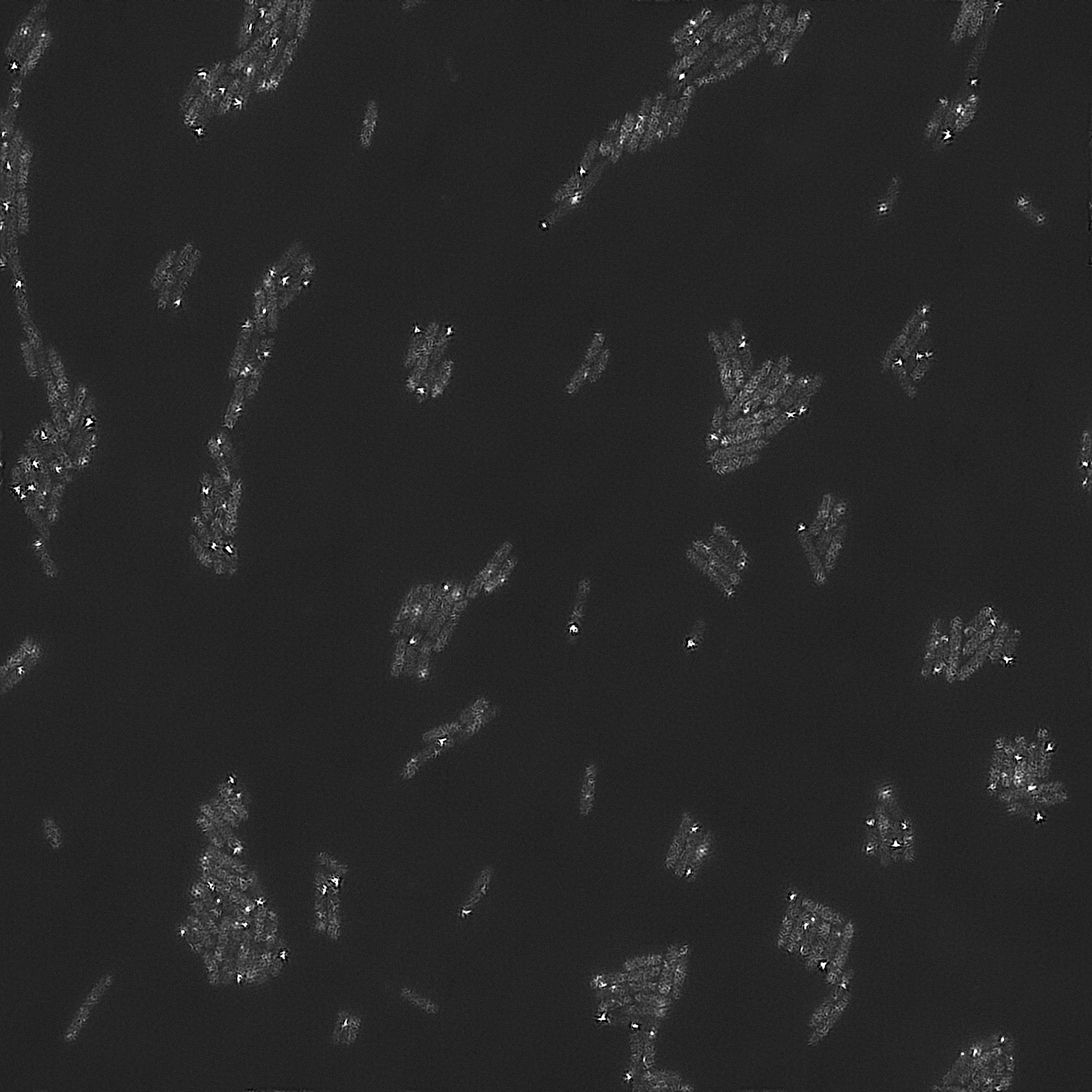

Supplement: Supplementary file 13 — Source Data [file 41467_2024_47330_MOESM13_ESM.zip › Source Data/Figure_6bc/mCherry/mCherry_01.tif]

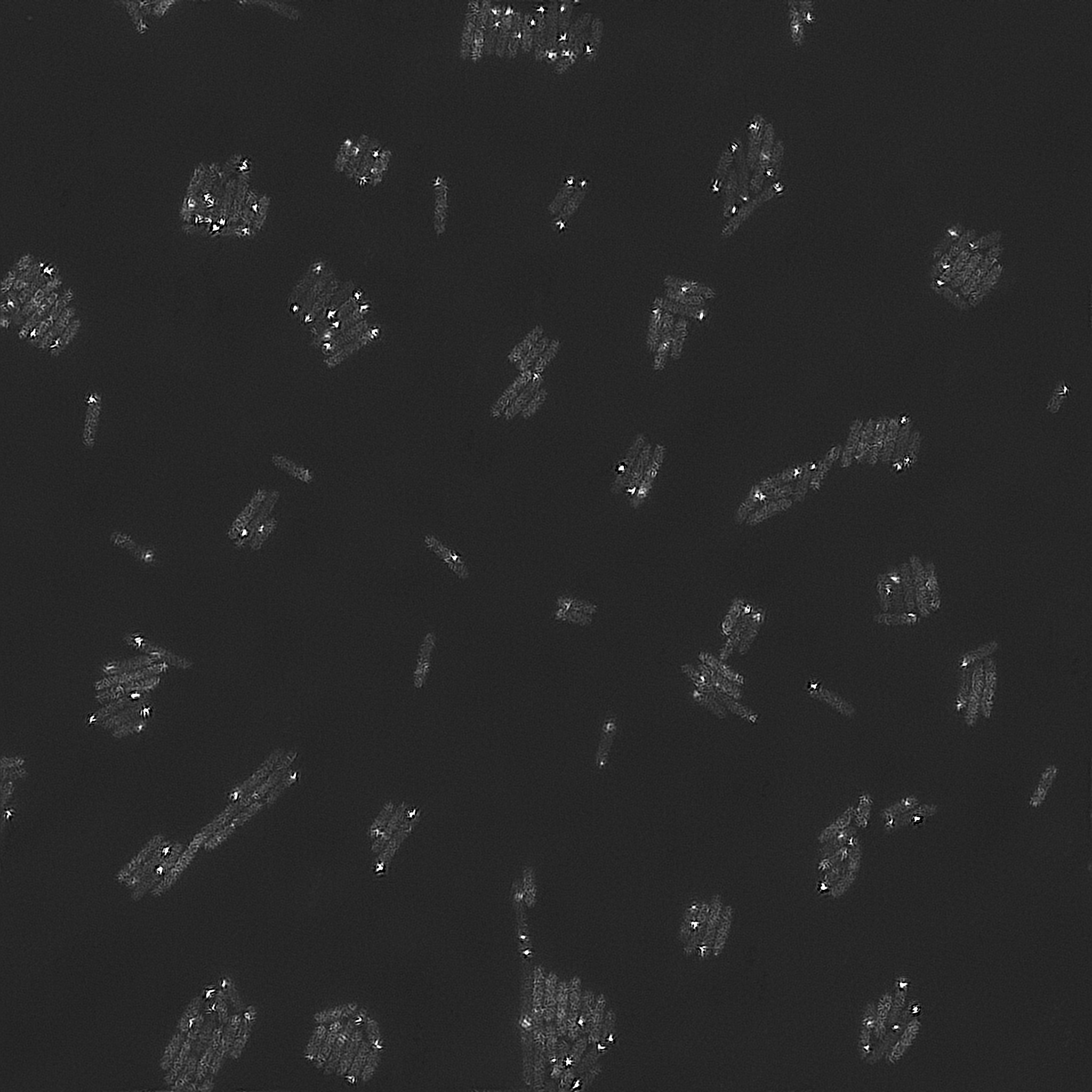

Supplement: Supplementary file 13 — Source Data [file 41467_2024_47330_MOESM13_ESM.zip › Source Data/Figure_6bc/mCherry/mCherry_02.tif]

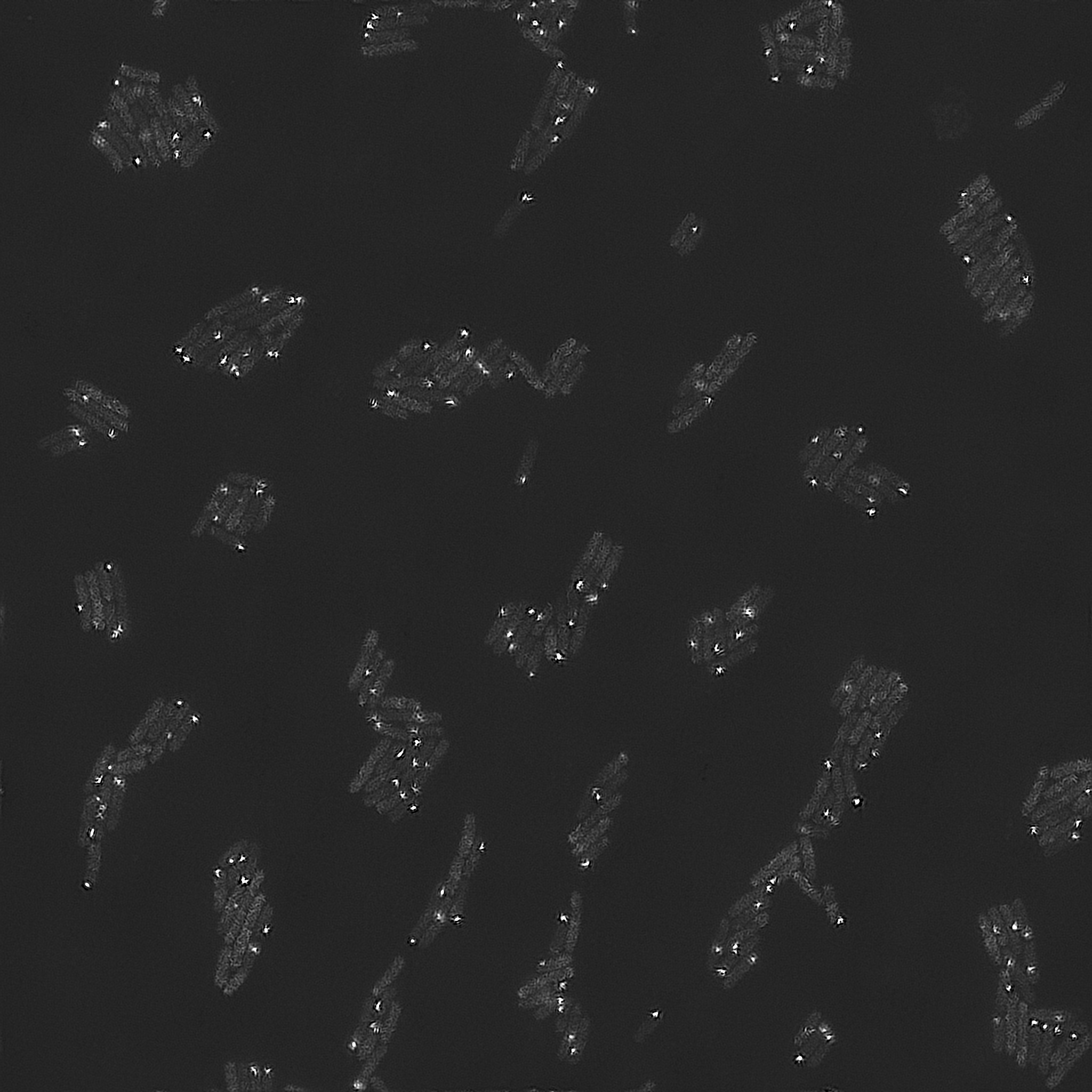

Supplement: Supplementary file 13 — Source Data [file 41467_2024_47330_MOESM13_ESM.zip › Source Data/Figure_6bc/mCherry/mCherry_03.tif]

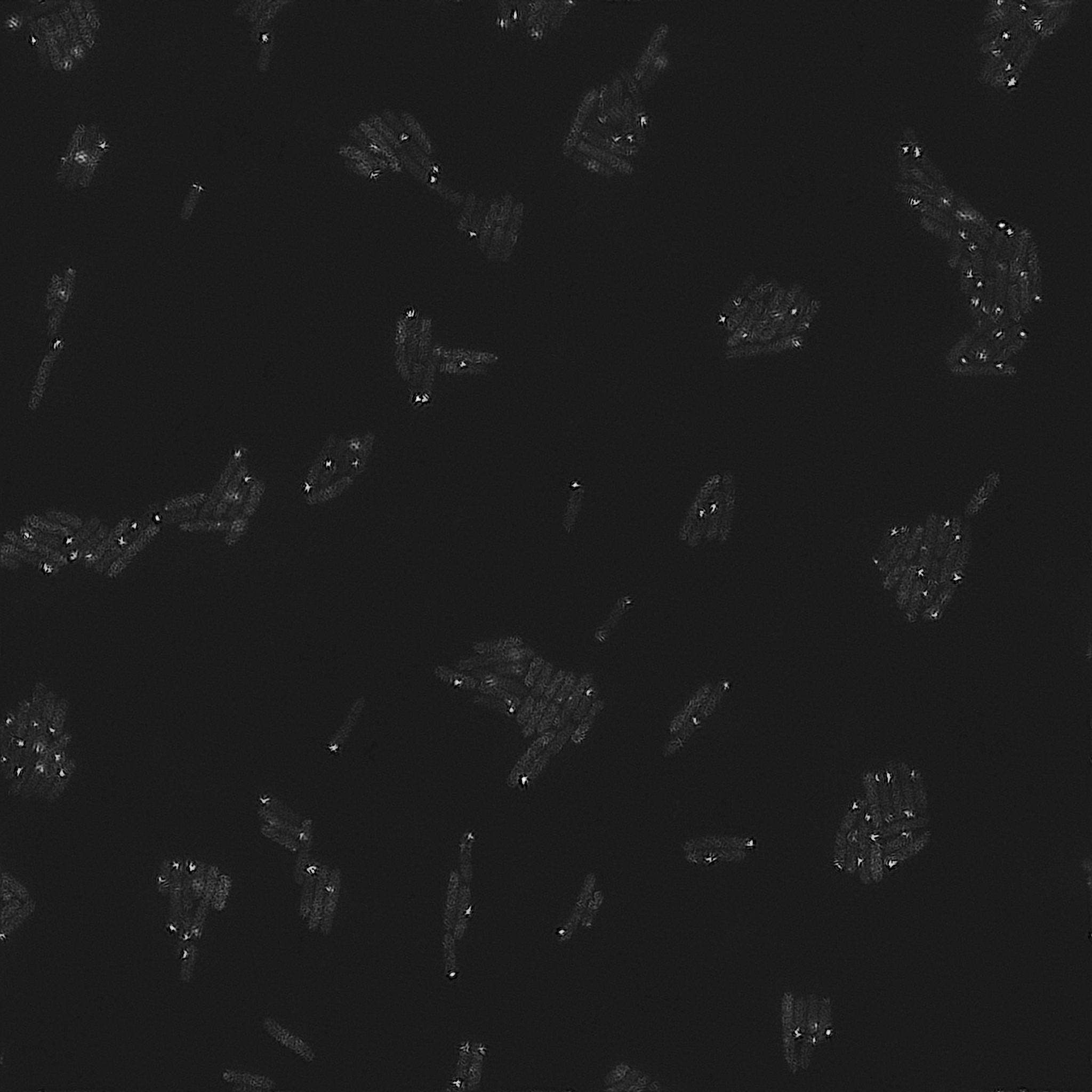

Supplement: Supplementary file 13 — Source Data [file 41467_2024_47330_MOESM13_ESM.zip › Source Data/Figure_6bc/mCherry/mCherry_04.tif]

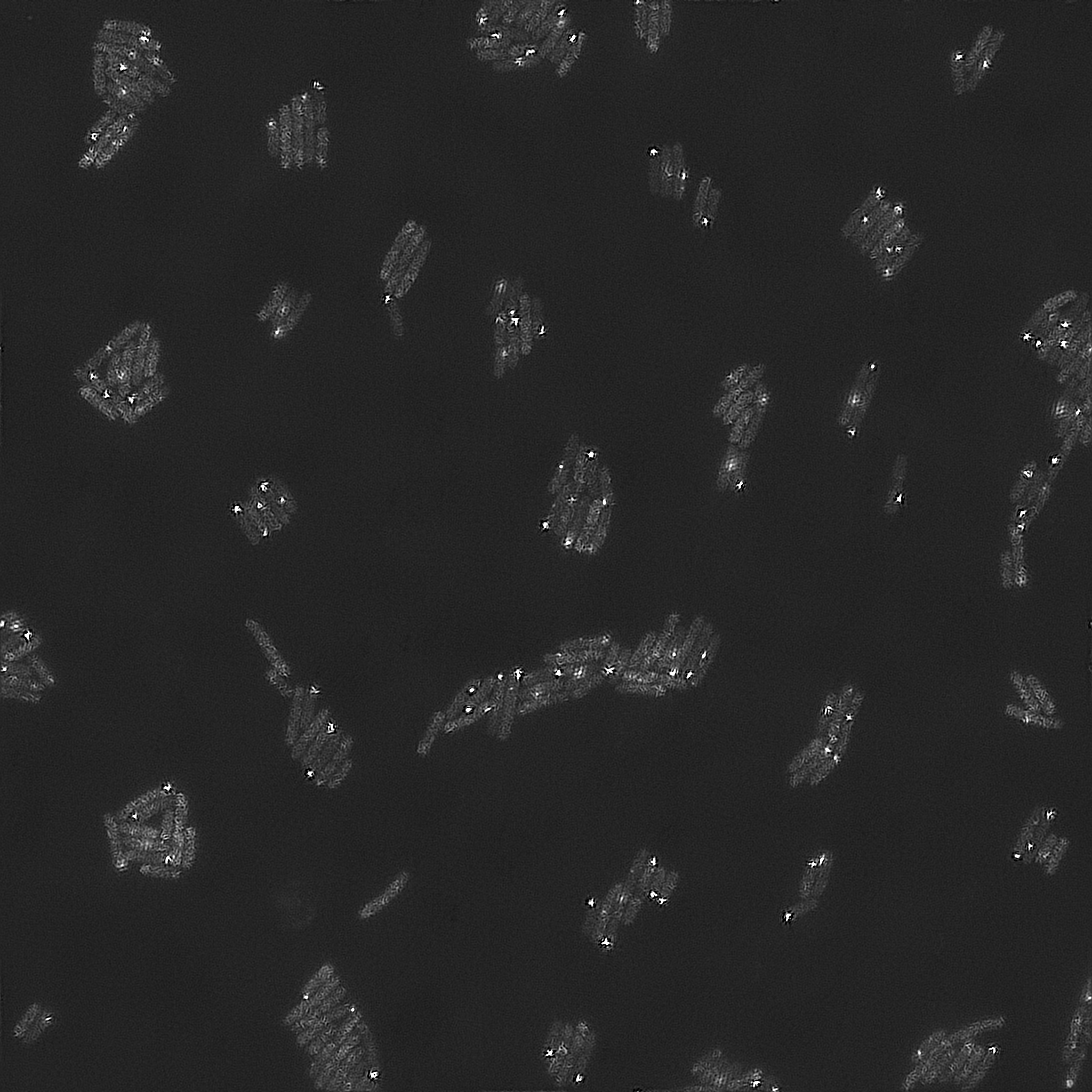

Supplement: Supplementary file 13 — Source Data [file 41467_2024_47330_MOESM13_ESM.zip › Source Data/Figure_6bc/mCherry/mCherry_05.tif]

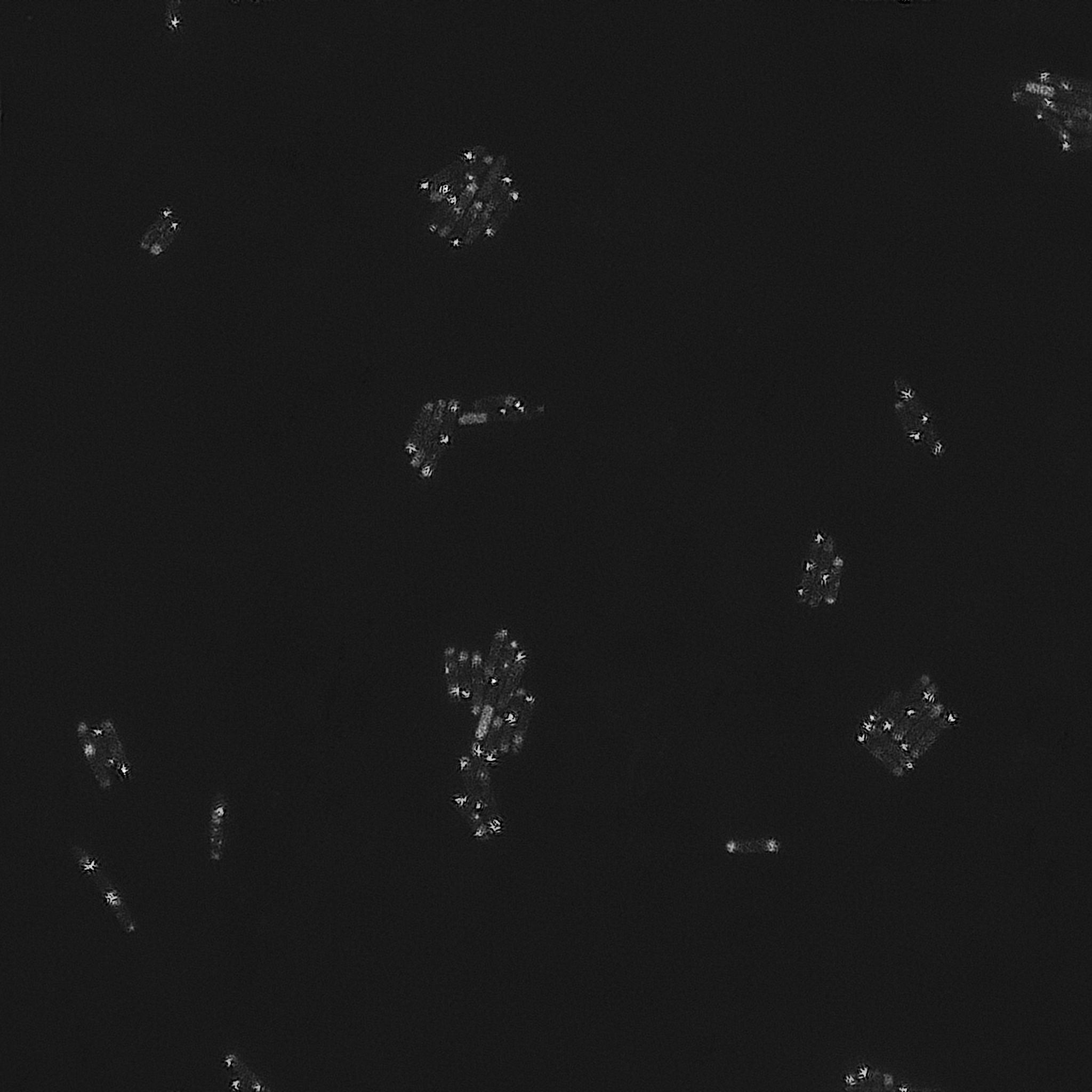

Supplement: Supplementary file 13 — Source Data [file 41467_2024_47330_MOESM13_ESM.zip › Source Data/Figure_6bc/PopTag_LL/pop78_01.tif]

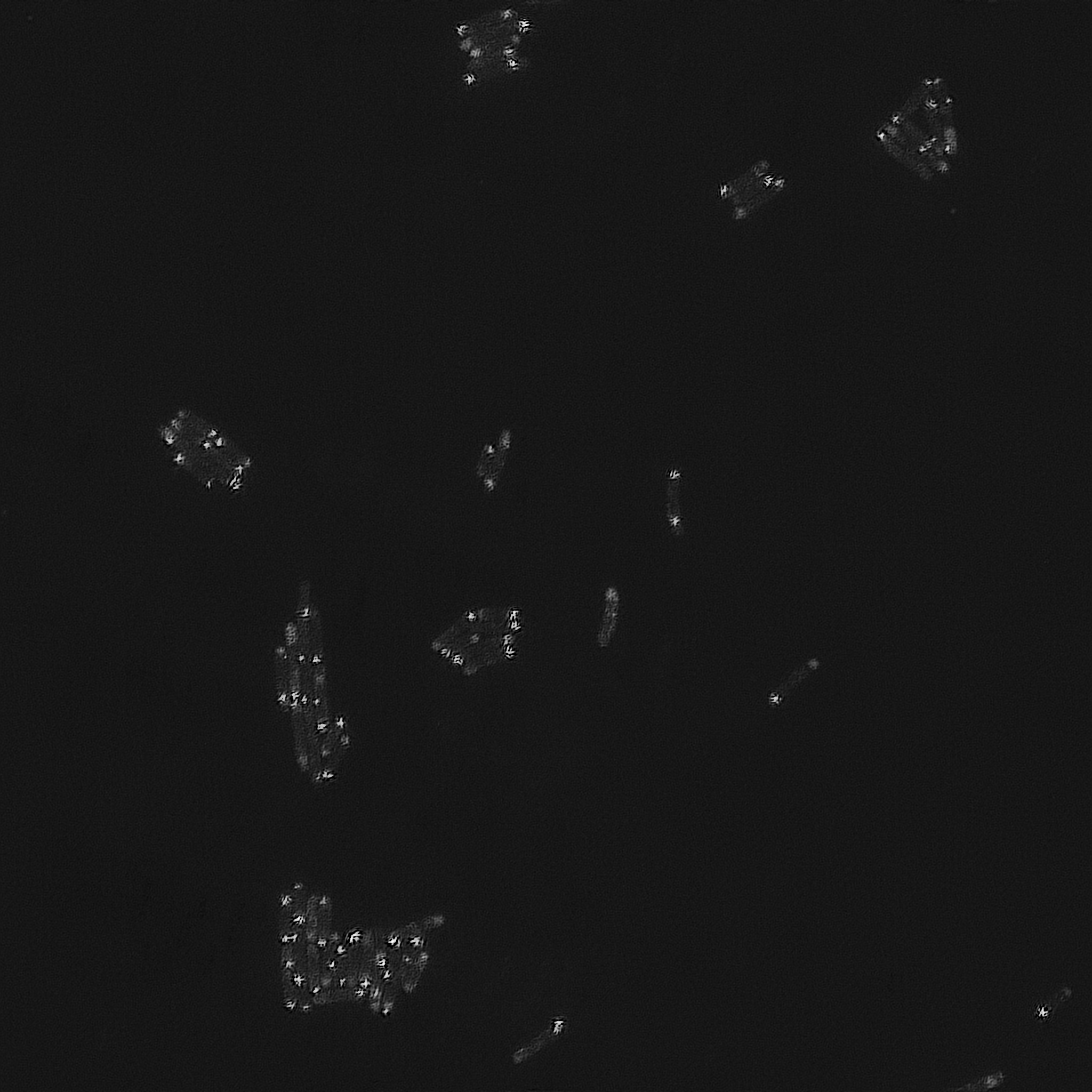

Supplement: Supplementary file 13 — Source Data [file 41467_2024_47330_MOESM13_ESM.zip › Source Data/Figure_6bc/PopTag_LL/pop78_02.tif]

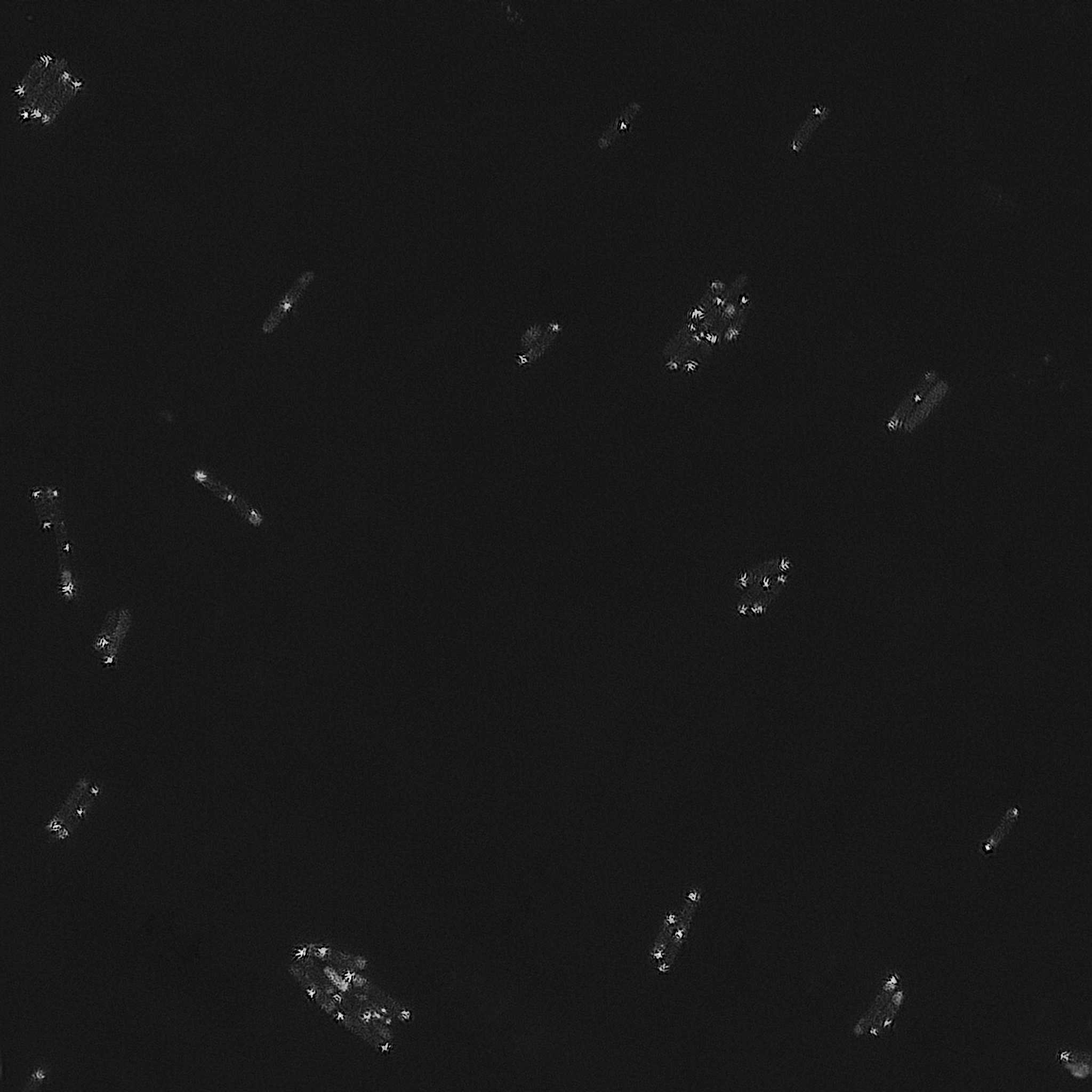

Supplement: Supplementary file 13 — Source Data [file 41467_2024_47330_MOESM13_ESM.zip › Source Data/Figure_6bc/PopTag_LL/pop78_03.tif]

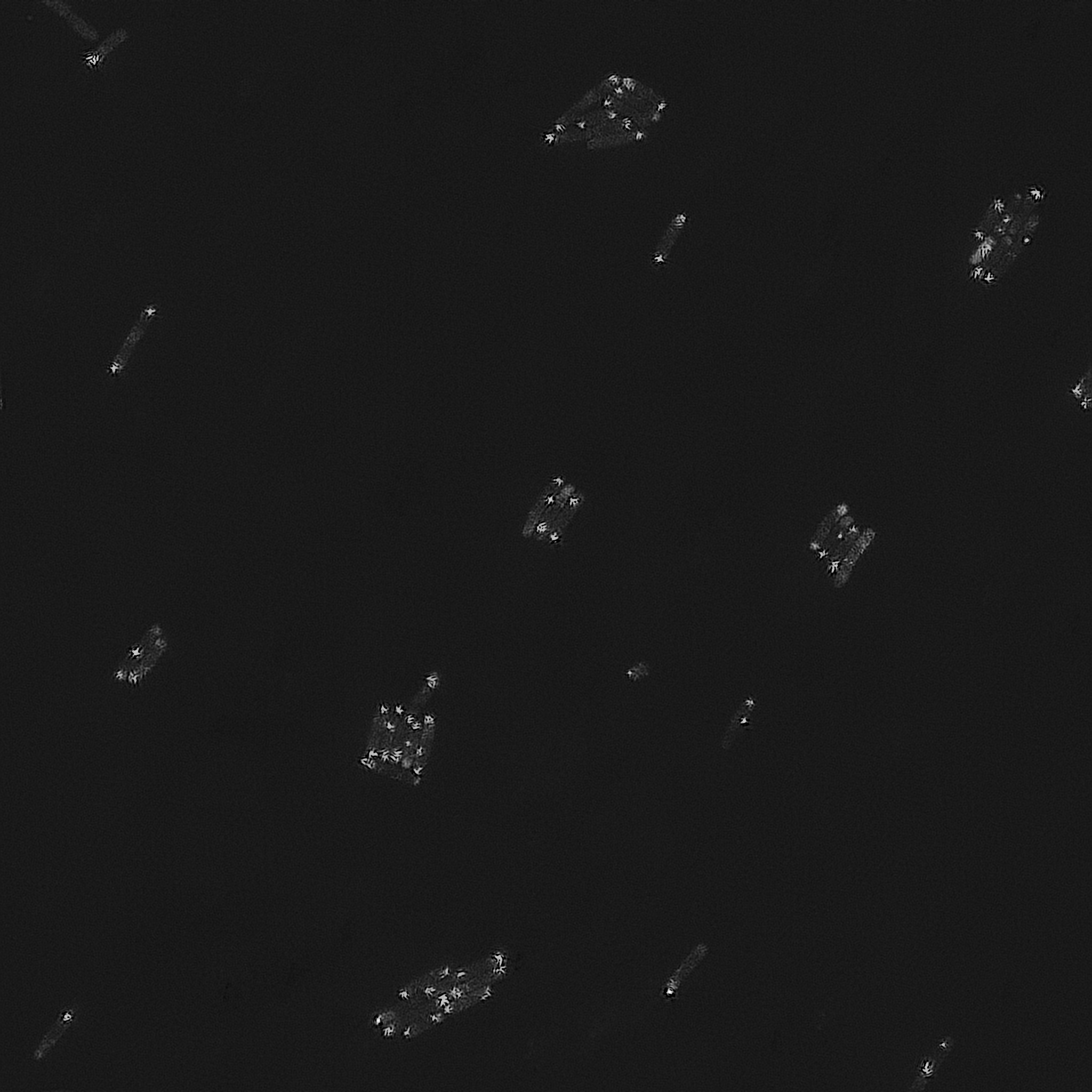

Supplement: Supplementary file 13 — Source Data [file 41467_2024_47330_MOESM13_ESM.zip › Source Data/Figure_6bc/PopTag_LL/pop78_04.tif]

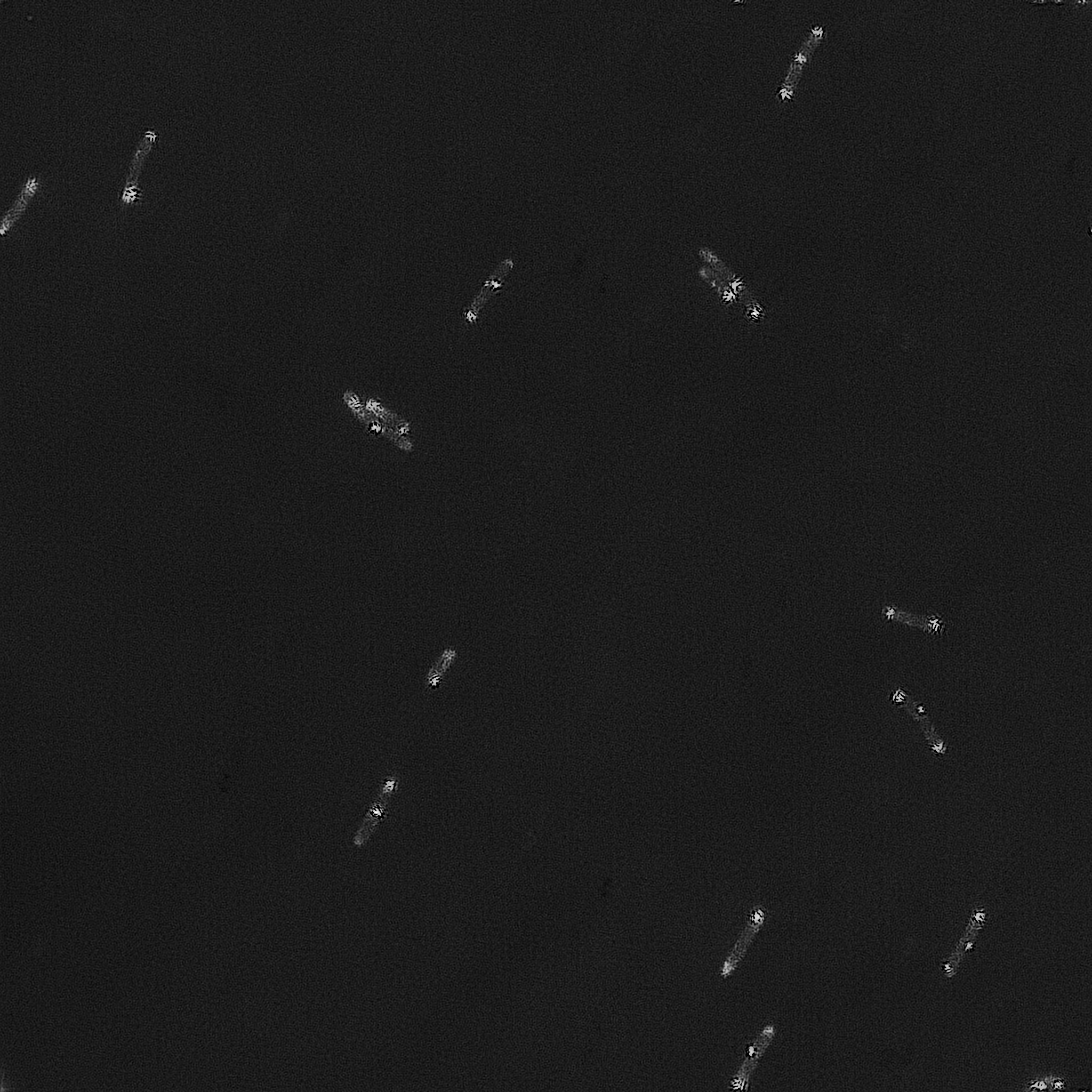

Supplement: Supplementary file 13 — Source Data [file 41467_2024_47330_MOESM13_ESM.zip › Source Data/Figure_6bc/PopTag_LL/pop78_05.tif]

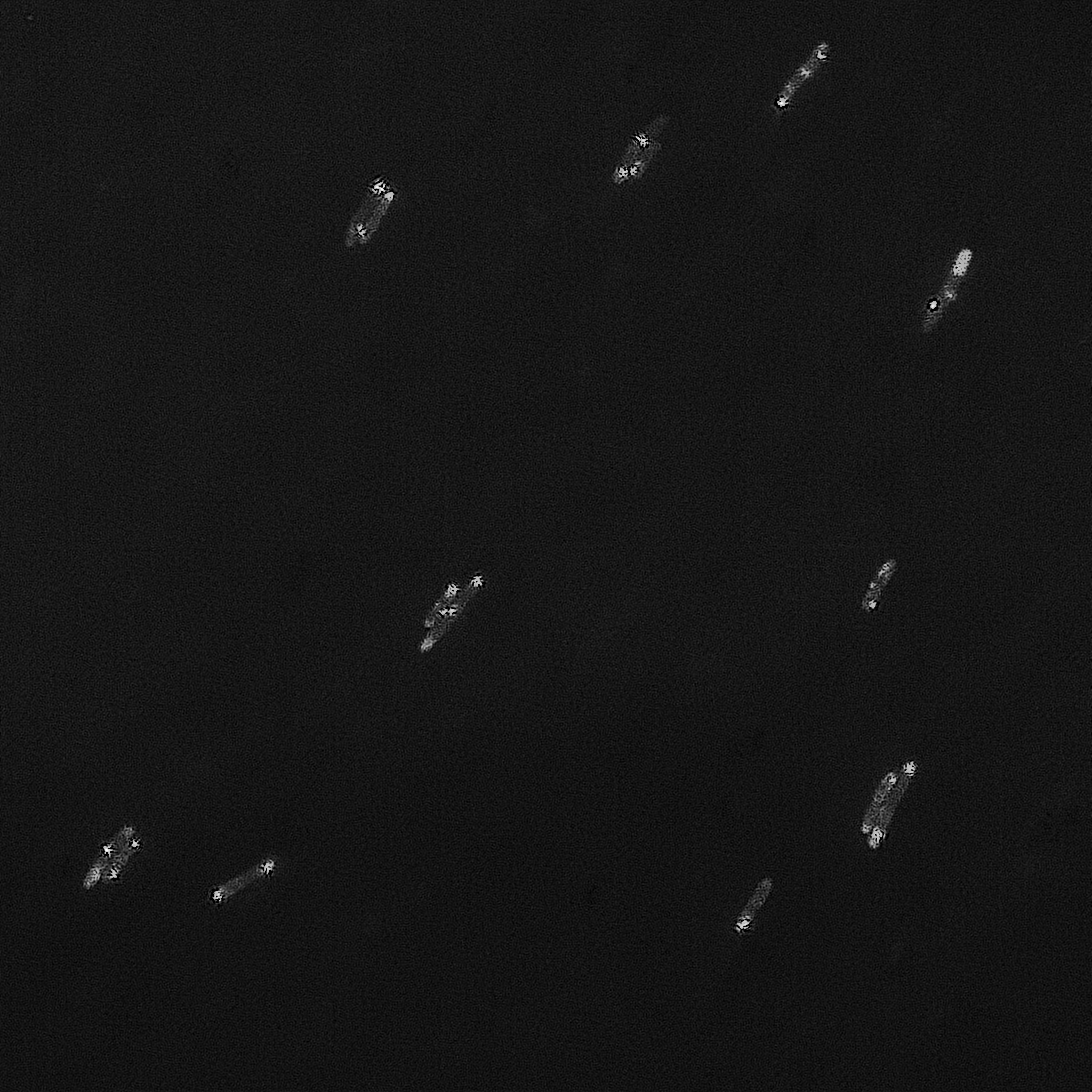

Supplement: Supplementary file 13 — Source Data [file 41467_2024_47330_MOESM13_ESM.zip › Source Data/Figure_6bc/PopTag_LL/pop78_06.tif]

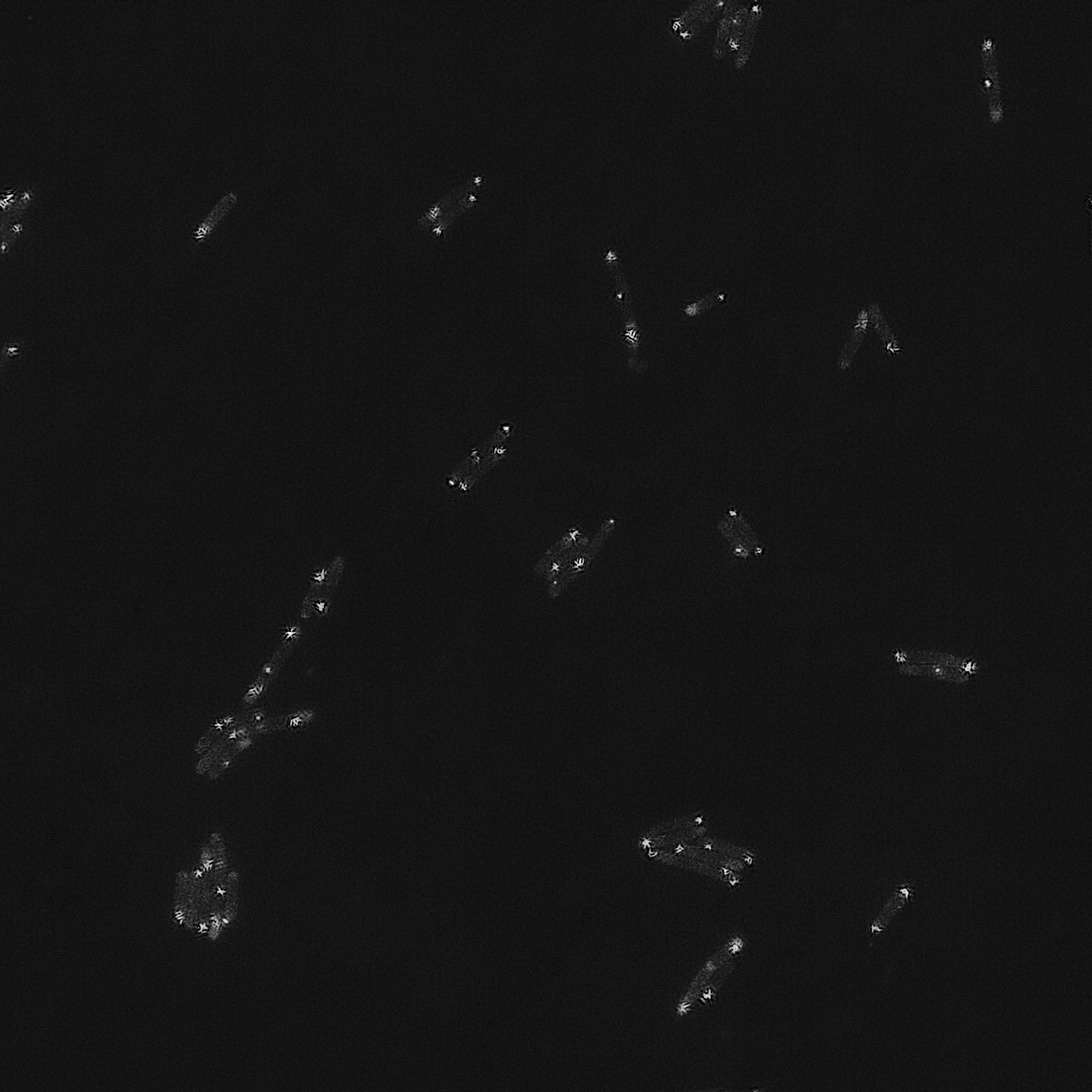

Supplement: Supplementary file 13 — Source Data [file 41467_2024_47330_MOESM13_ESM.zip › Source Data/Figure_6bc/PopTag_LL/pop78_07.tif]

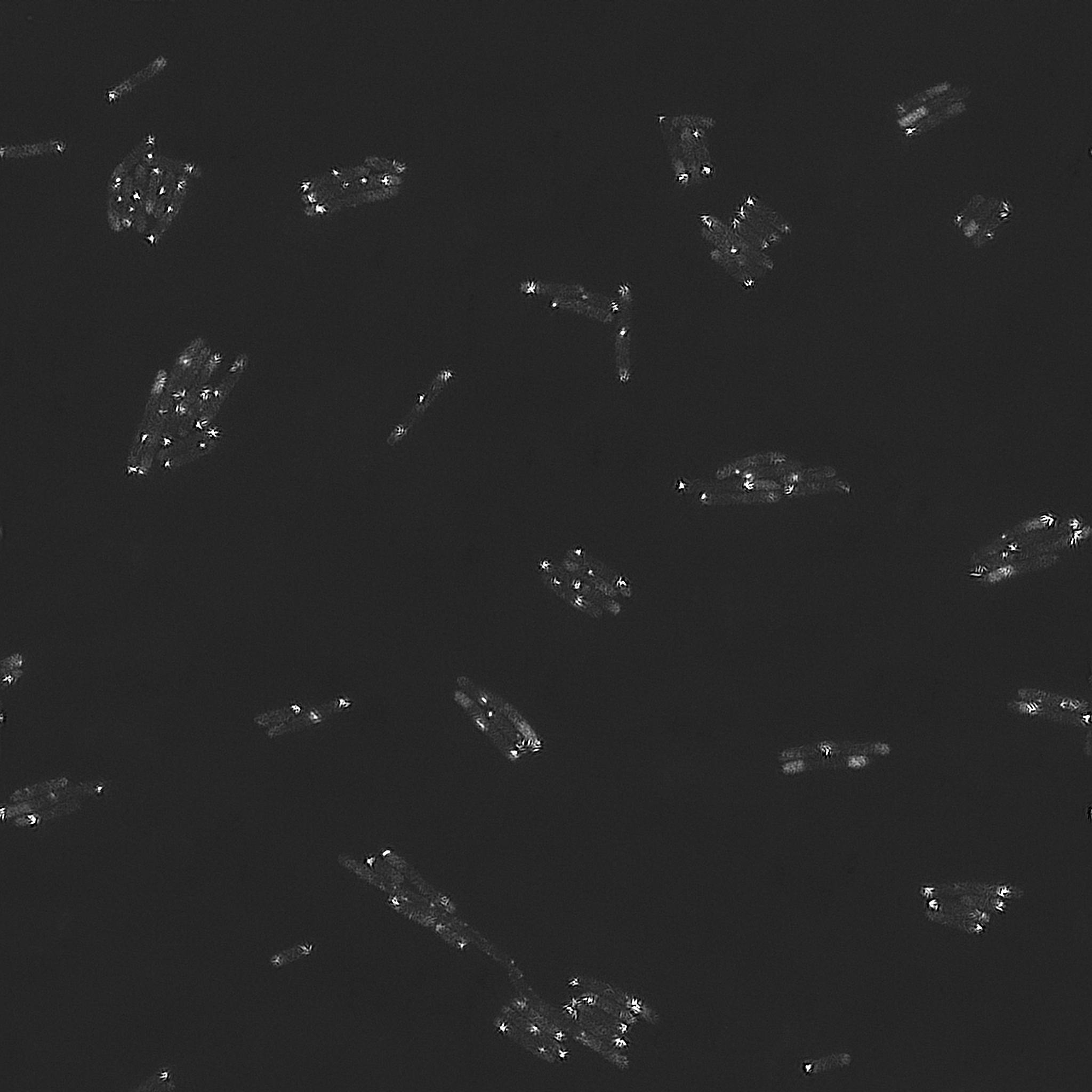

Supplement: Supplementary file 13 — Source Data [file 41467_2024_47330_MOESM13_ESM.zip › Source Data/Figure_6bc/PopTag_LL/pop78_08.tif]

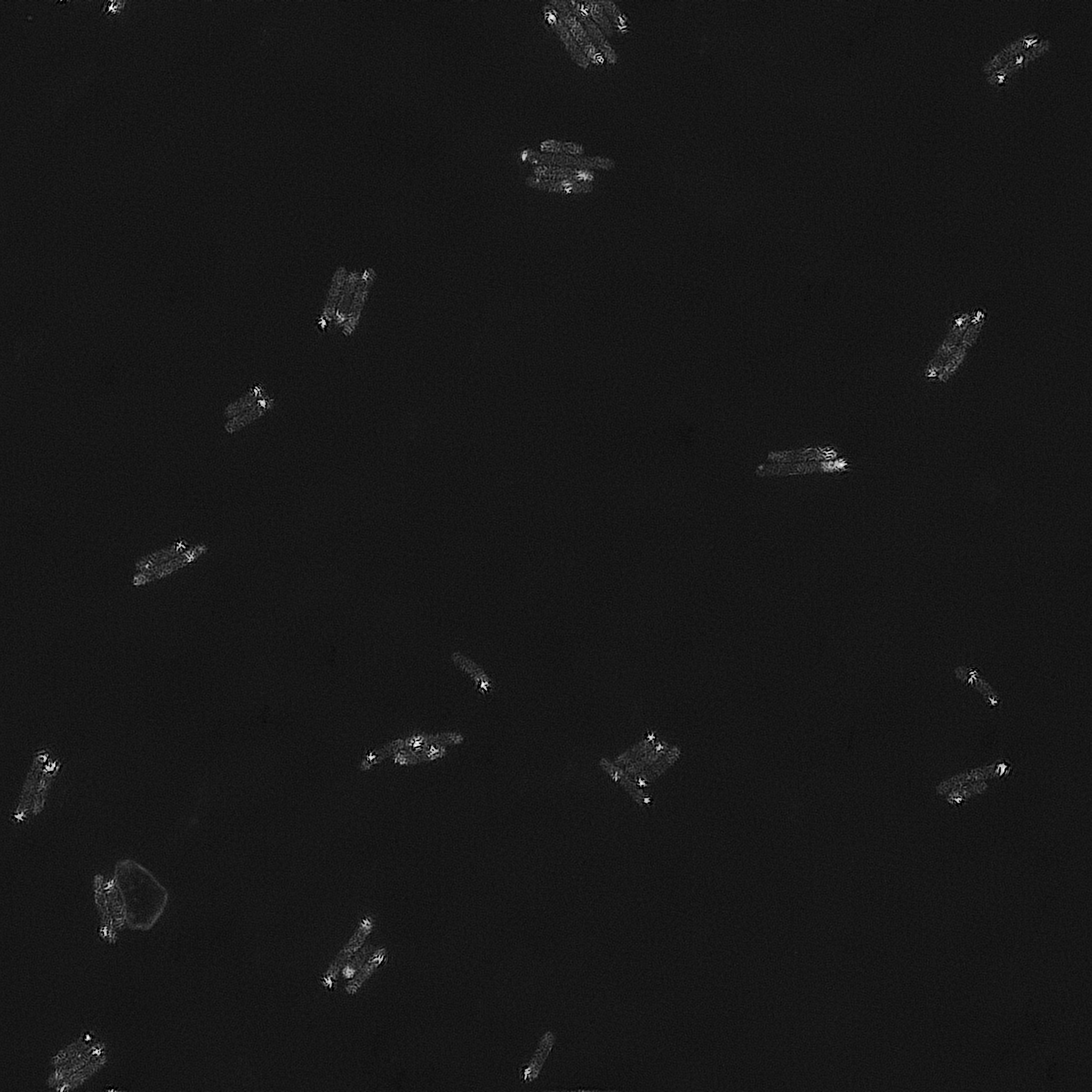

Supplement: Supplementary file 13 — Source Data [file 41467_2024_47330_MOESM13_ESM.zip › Source Data/Figure_6bc/PopTag_LL/pop78_09.tif]

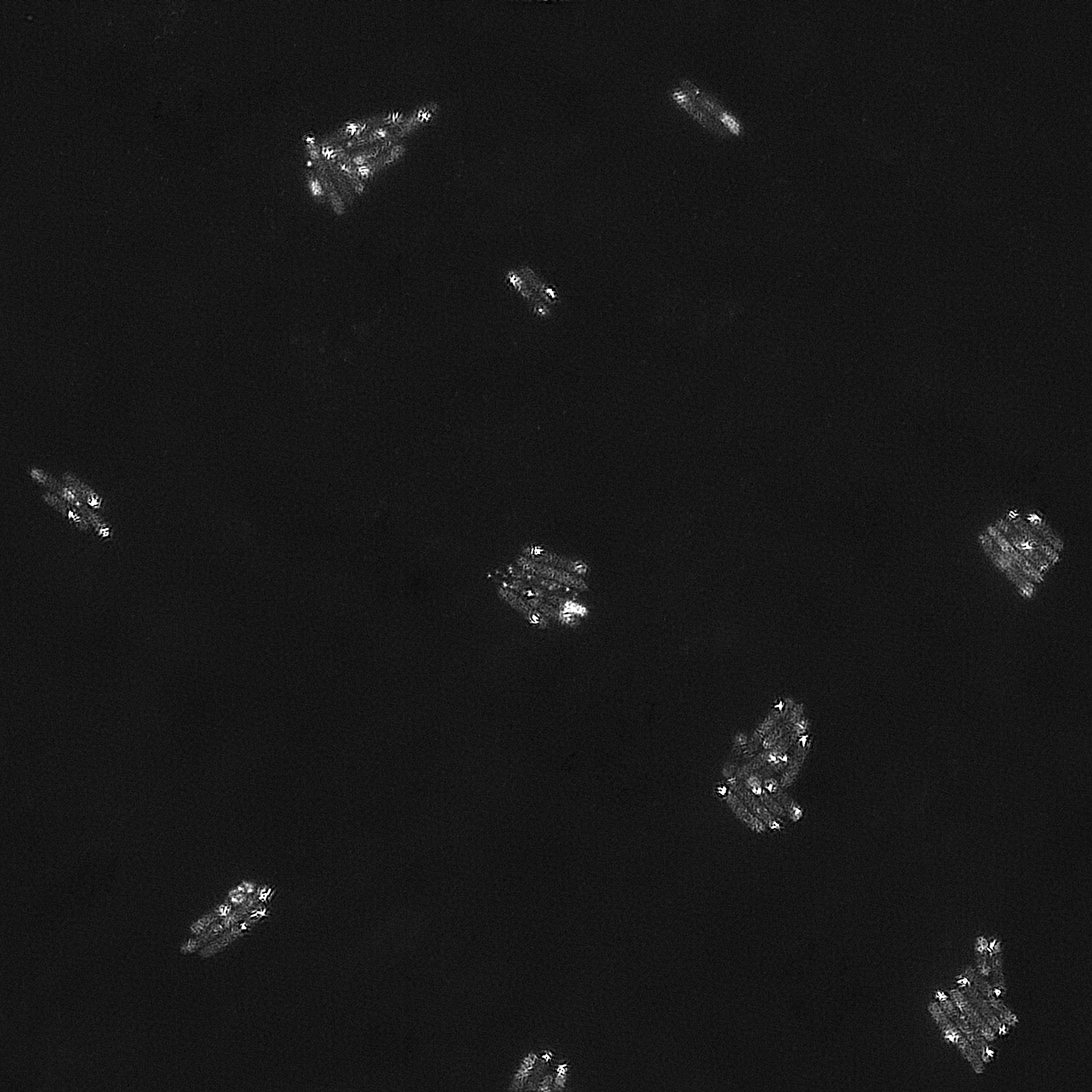

Supplement: Supplementary file 13 — Source Data [file 41467_2024_47330_MOESM13_ESM.zip › Source Data/Figure_6bc/PopTag_LL/pop78_10.tif]

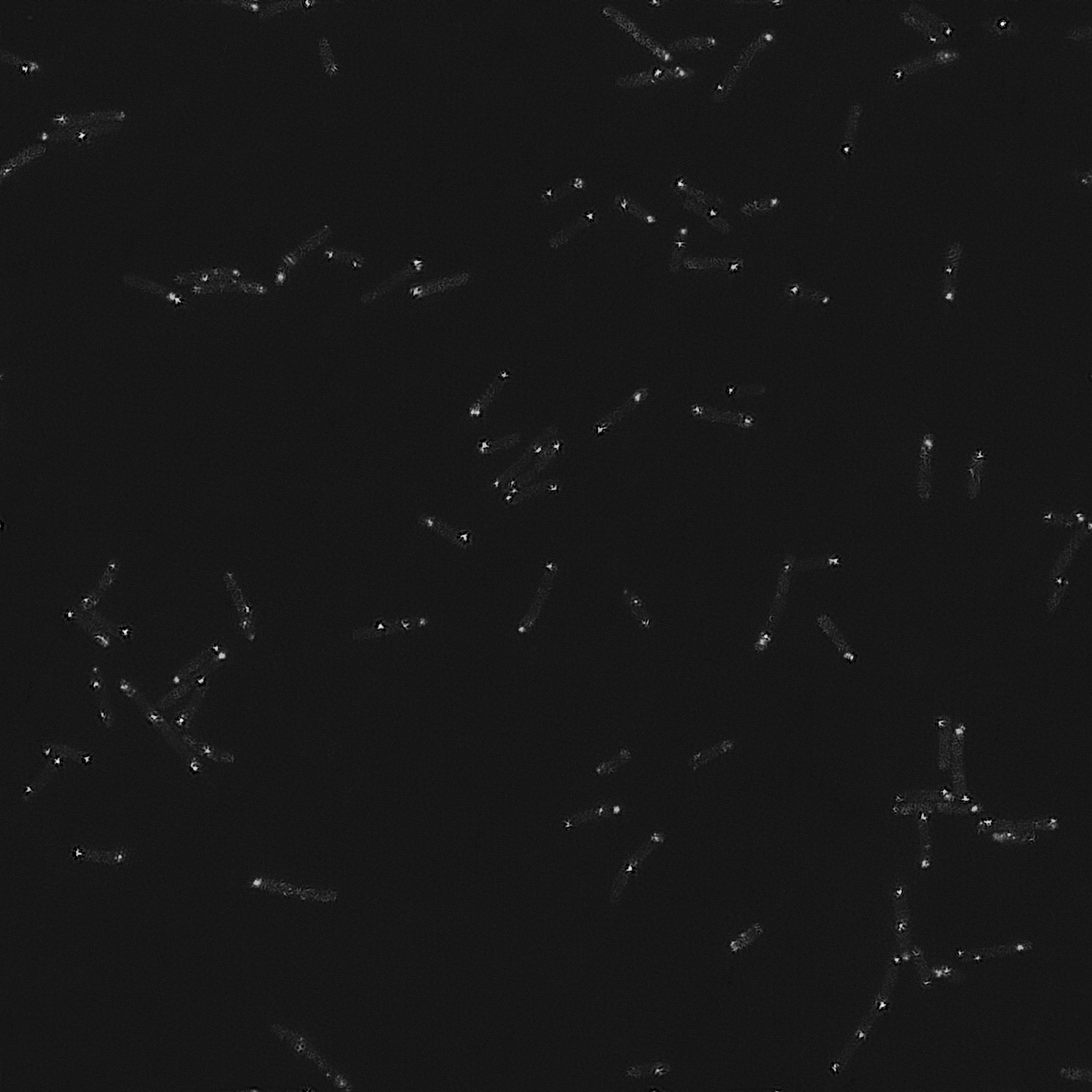

Supplement: Supplementary file 13 — Source Data [file 41467_2024_47330_MOESM13_ESM.zip › Source Data/Figure_6bc/PopTag_SL/pop6_01.tif]

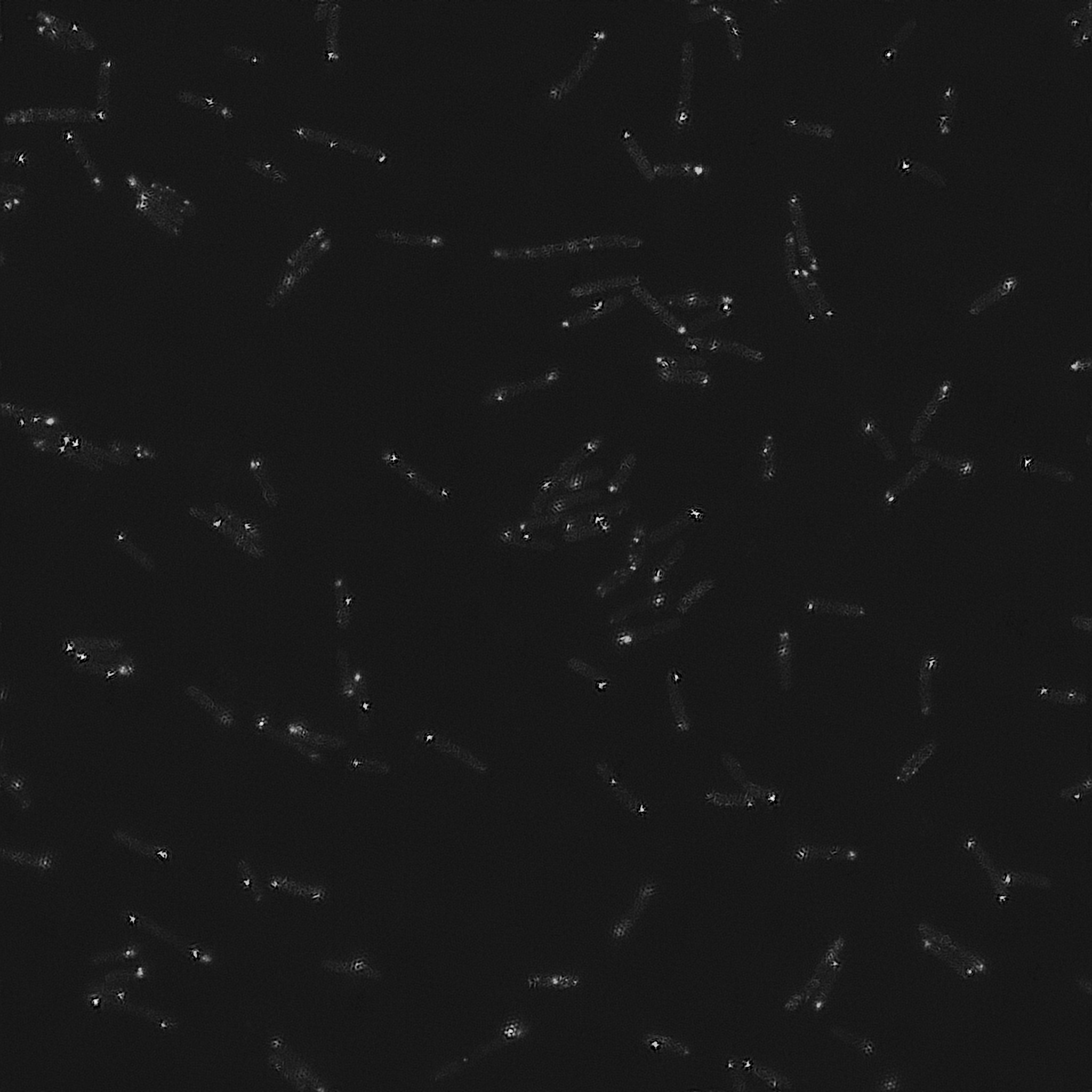

Supplement: Supplementary file 13 — Source Data [file 41467_2024_47330_MOESM13_ESM.zip › Source Data/Figure_6bc/PopTag_SL/pop6_02.tif]

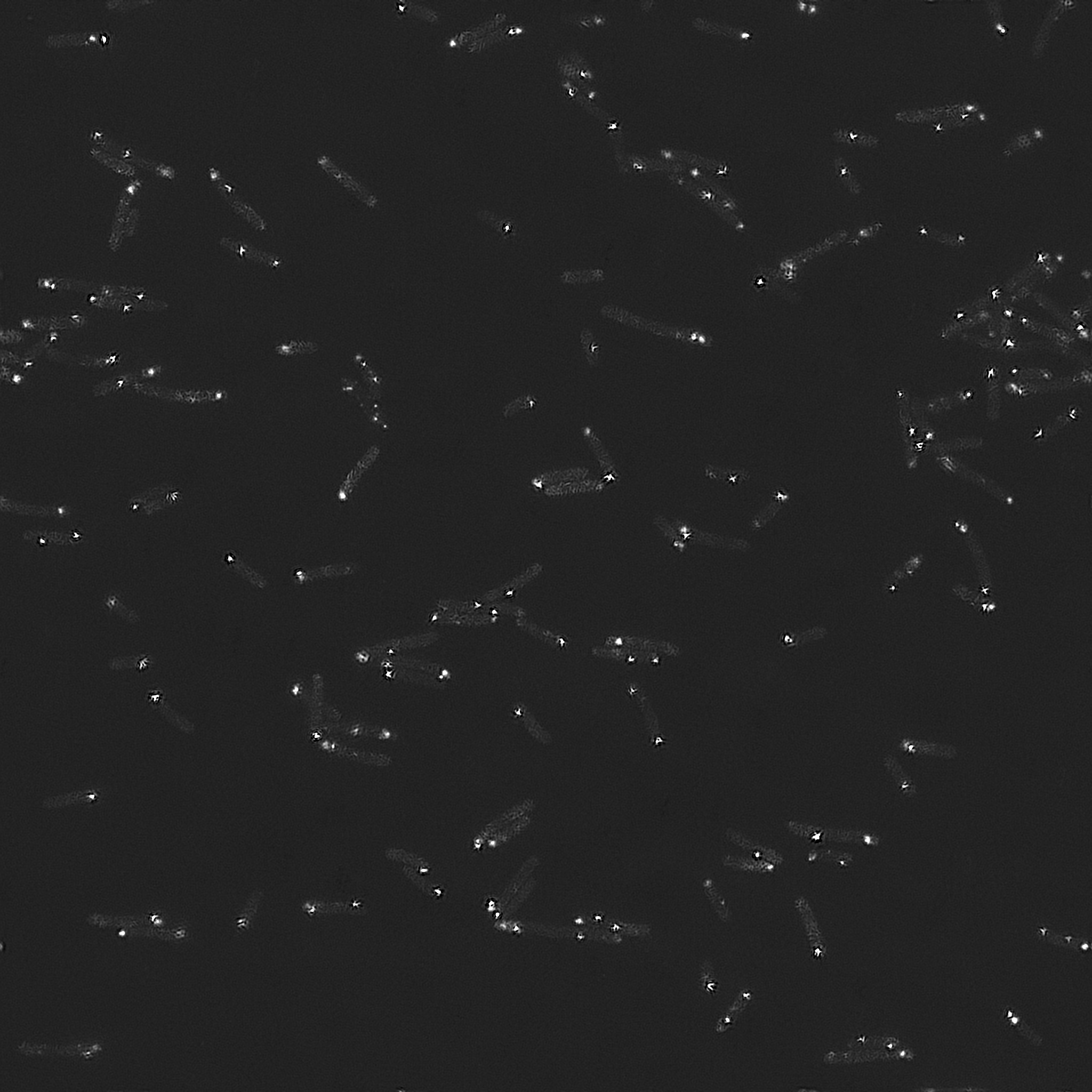

Supplement: Supplementary file 13 — Source Data [file 41467_2024_47330_MOESM13_ESM.zip › Source Data/Figure_6bc/PopTag_SL/pop6_03.tif]

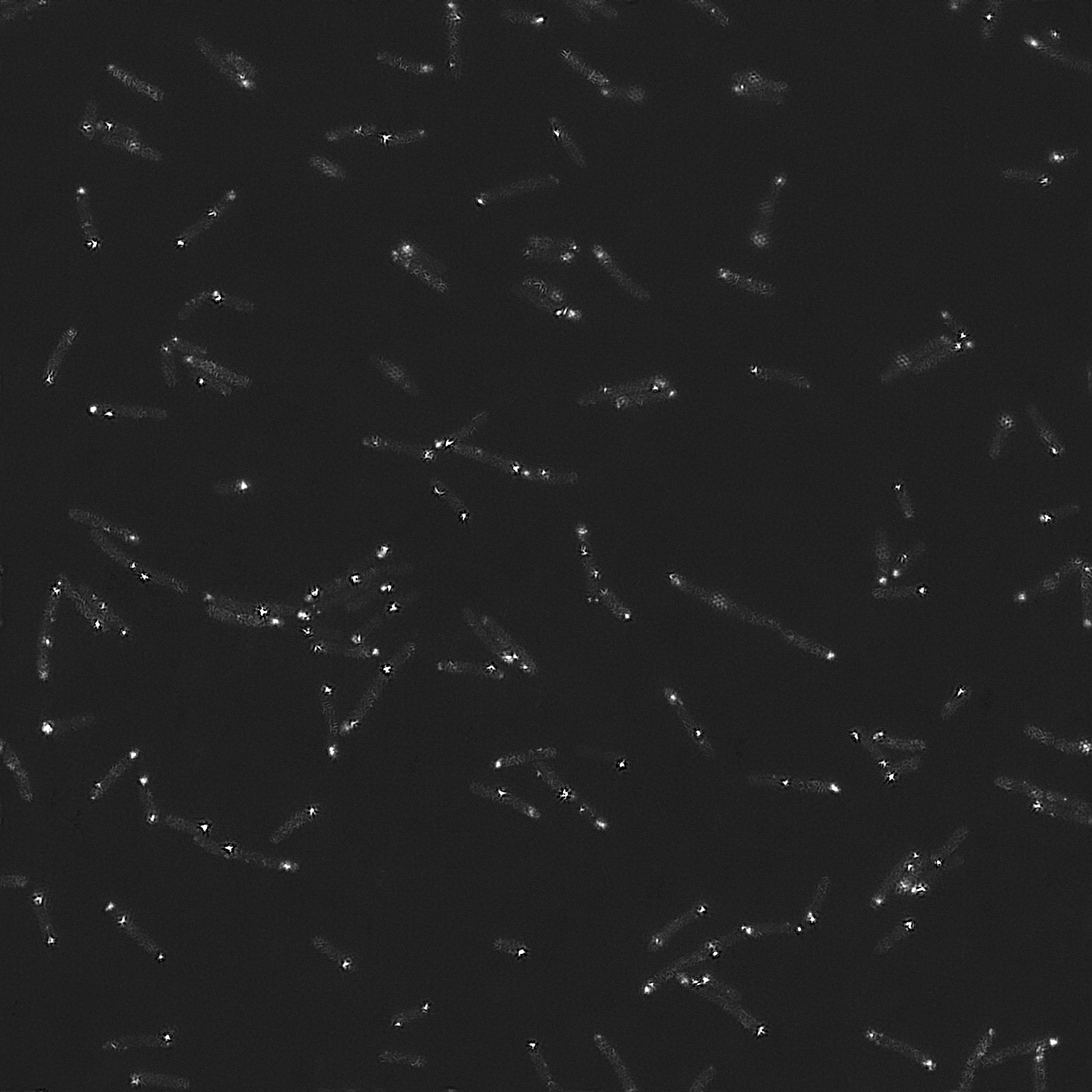

Supplement: Supplementary file 13 — Source Data [file 41467_2024_47330_MOESM13_ESM.zip › Source Data/Figure_6bc/PopTag_SL/pop6_04.tif]

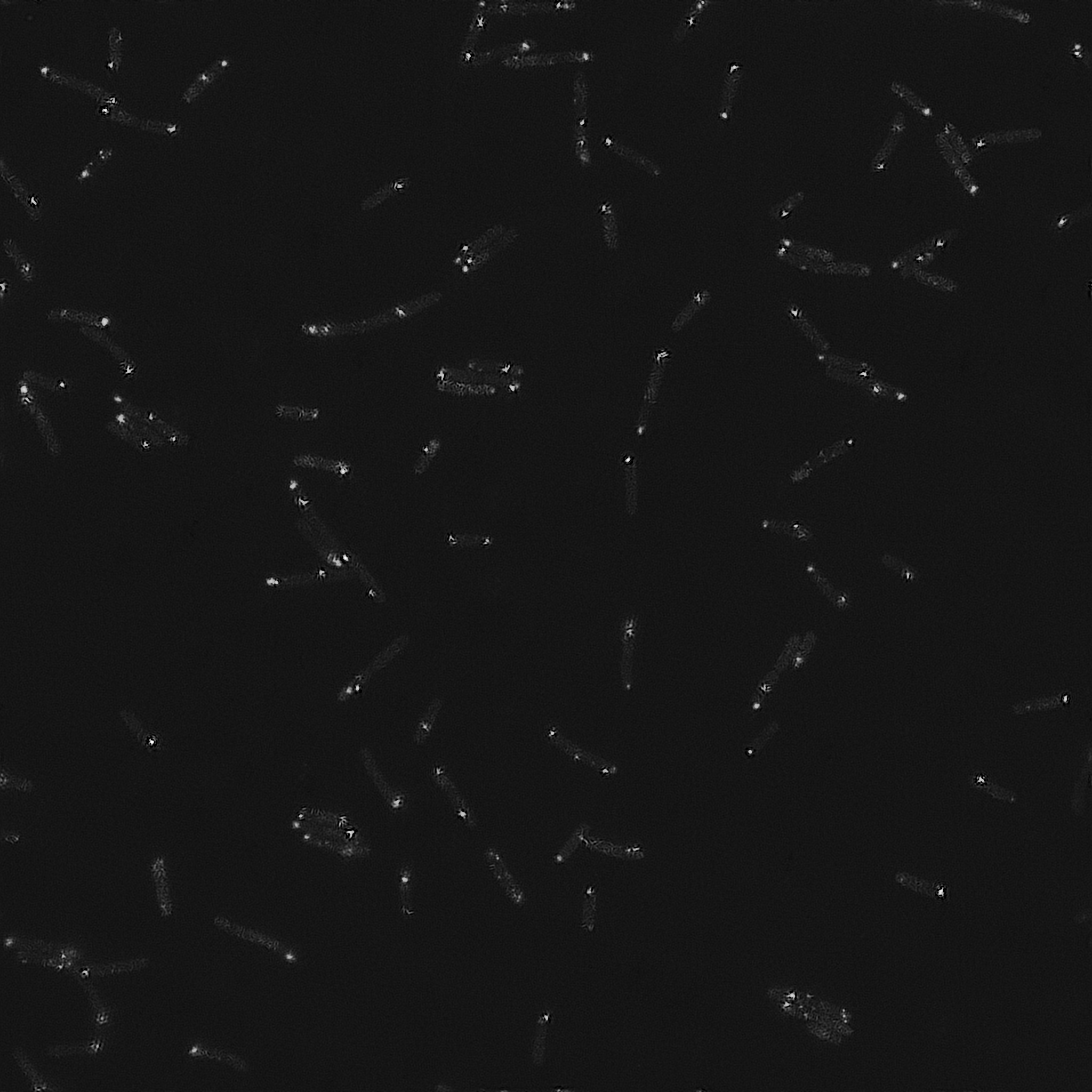

Supplement: Supplementary file 13 — Source Data [file 41467_2024_47330_MOESM13_ESM.zip › Source Data/Figure_6bc/PopTag_SL/pop6_05.tif]

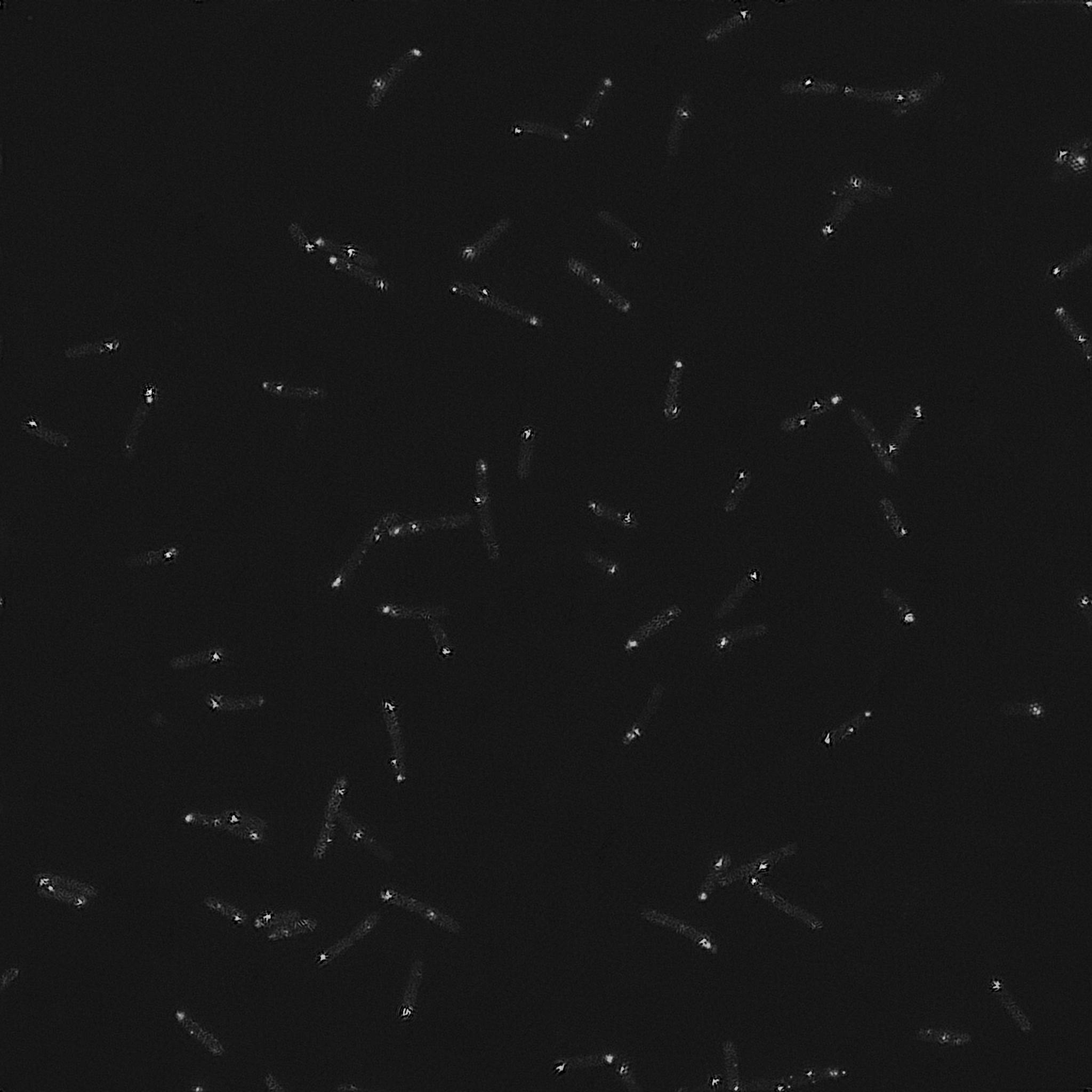

Supplement: Supplementary file 13 — Source Data [file 41467_2024_47330_MOESM13_ESM.zip › Source Data/Figure_6bc/PopTag_SL/pop6_06.tif]

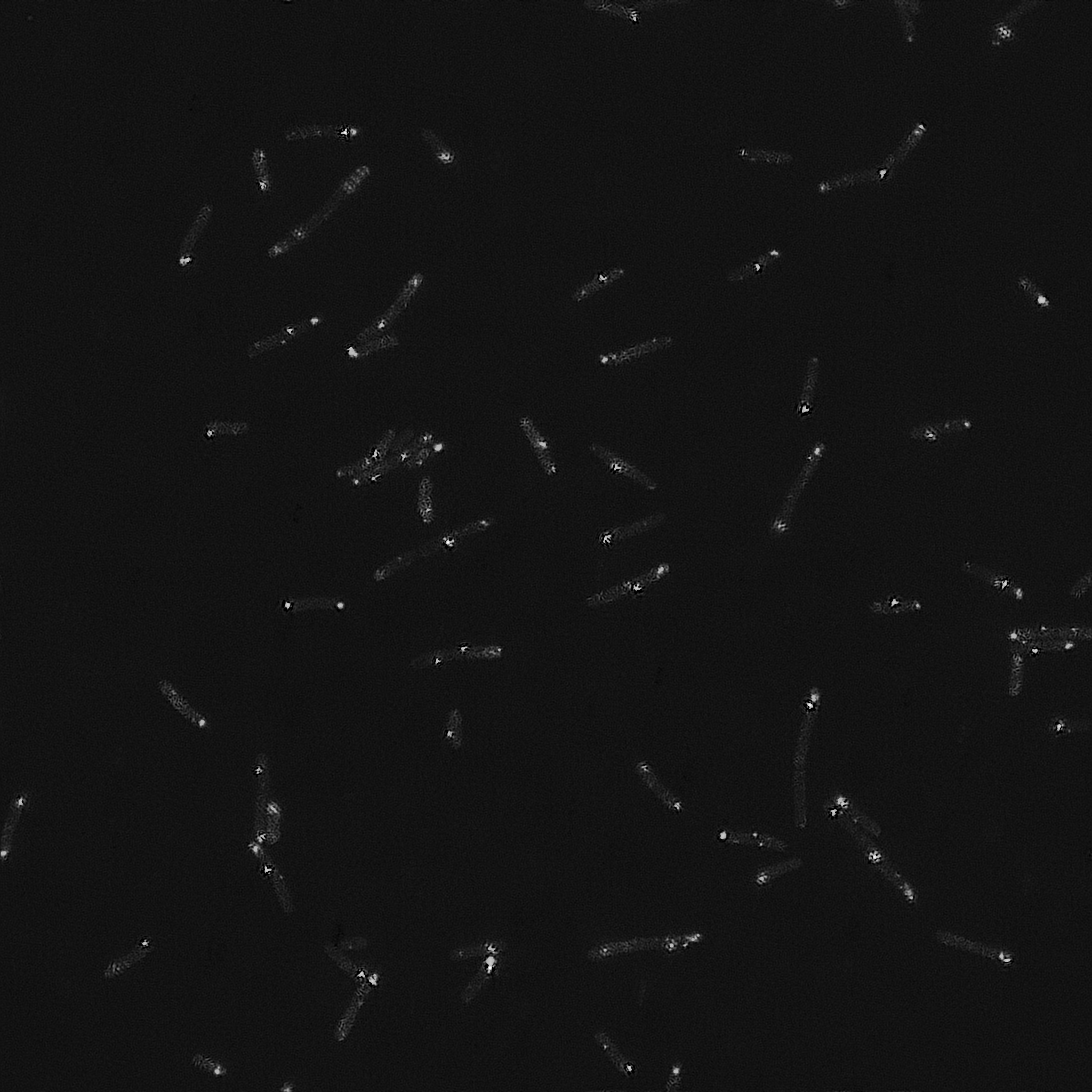

Supplement: Supplementary file 13 — Source Data [file 41467_2024_47330_MOESM13_ESM.zip › Source Data/Figure_6bc/PopTag_SL/pop6_07.tif]

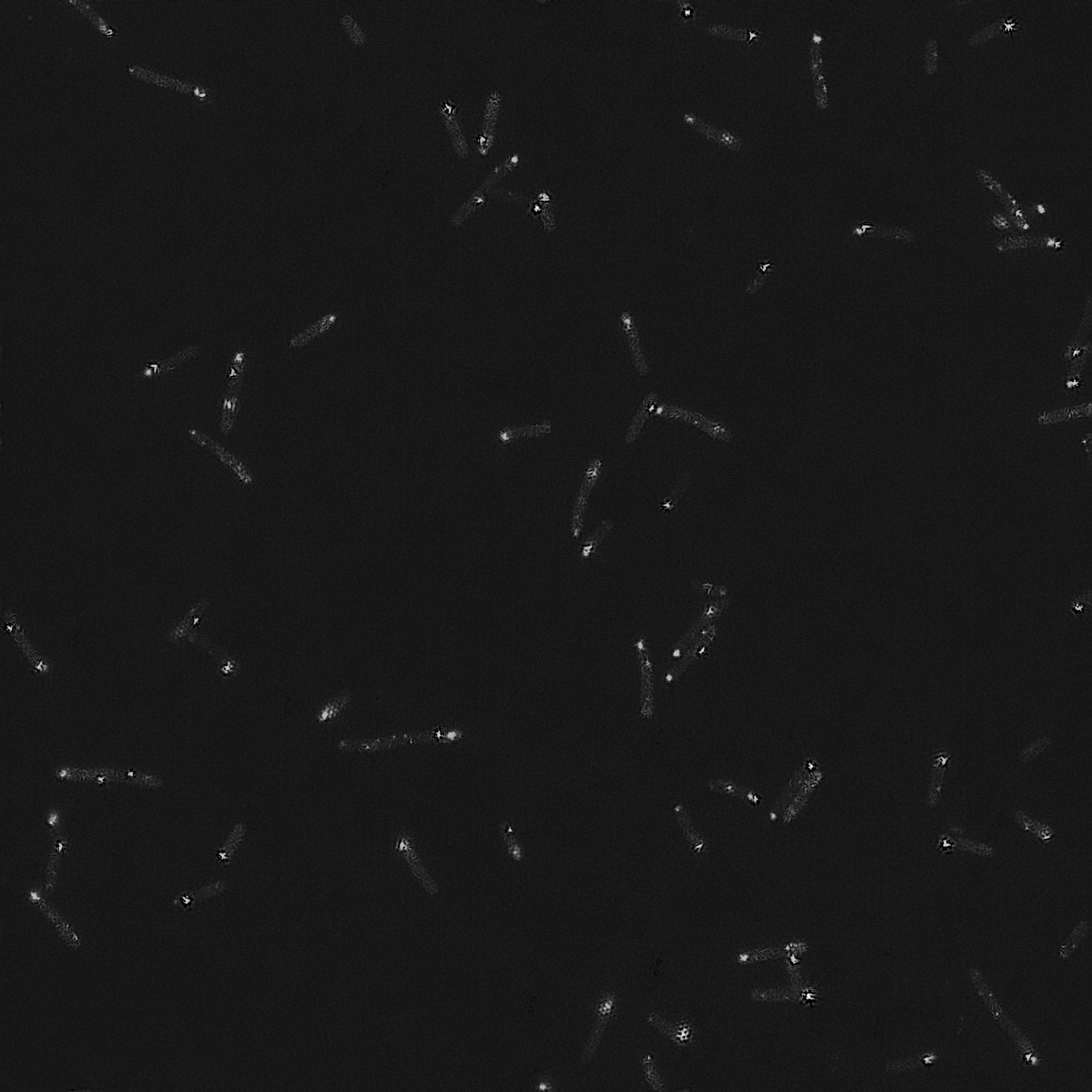

Supplement: Supplementary file 13 — Source Data [file 41467_2024_47330_MOESM13_ESM.zip › Source Data/Figure_6bc/PopTag_SL/pop6_08.tif]

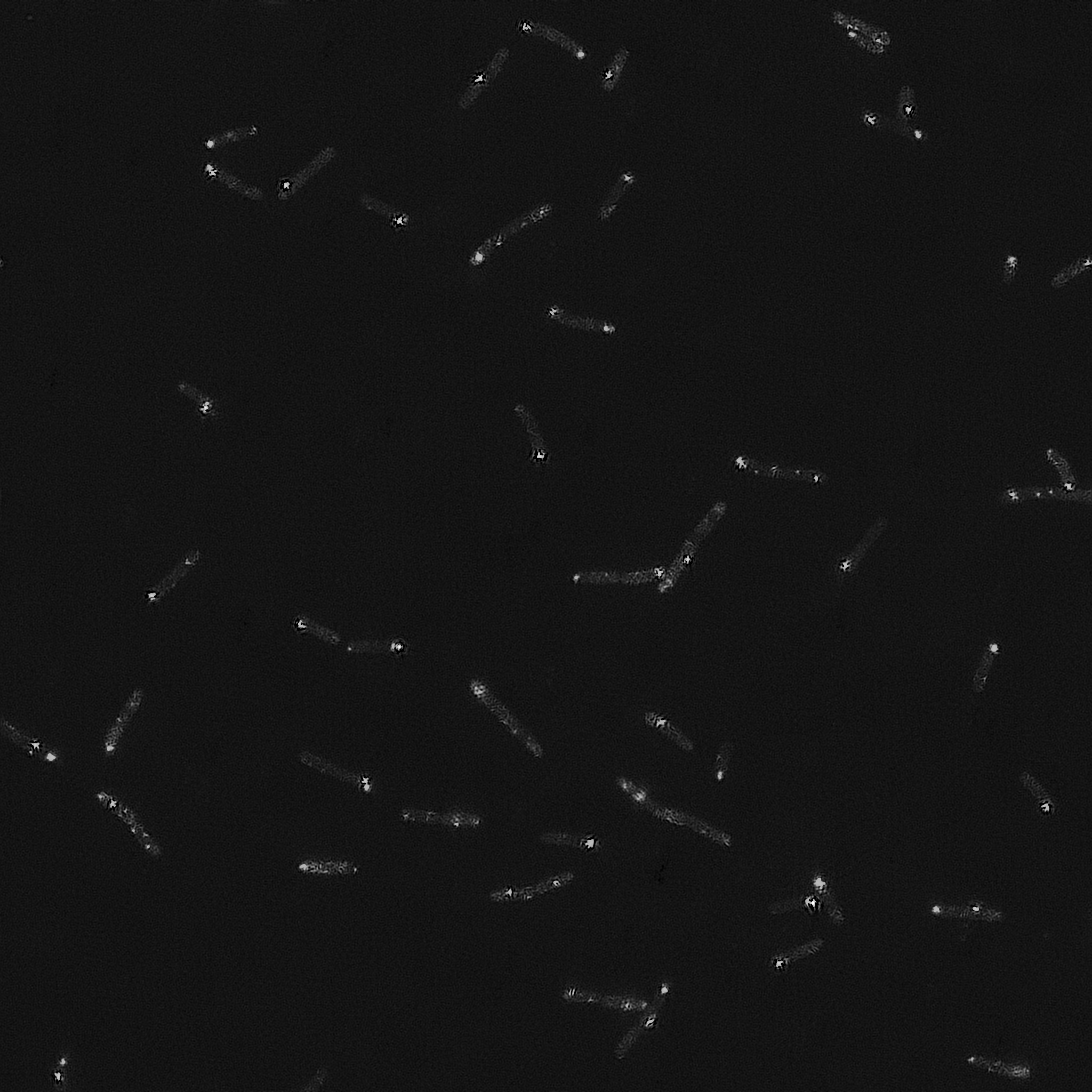

Supplement: Supplementary file 13 — Source Data [file 41467_2024_47330_MOESM13_ESM.zip › Source Data/Figure_6bc/PopTag_SL/pop6_09.tif]
